# Development and validation of a high-speed stereoscopic eyetracker

**DOI:** 10.3758/s13428-018-1026-7

**Published:** 2018-03-05

**Authors:** Annemiek D. Barsingerhorn, F. Nienke Boonstra, Jeroen Goossens

**Affiliations:** 1 Department of Cognitive Neuroscience, Biophysics Section, Donders Institute for Brain, Cognition and Behaviour, Radboud University Medical Centre Nijmegen, Nijmegen, The Netherlands; 2 Department of Biophysics, Donders Institute for Brain, Cognition and Behaviour, Radboud University Nijmegen, Nijmegen, The Netherlands; 3 Royal Dutch Visio—National Foundation for the Visually Impaired and Blind, Huizen, The Netherlands; 4 Bartiméus Institute for the Visually Impaired, Zeist, The Netherlands

**Keywords:** Eyetracking, Eye movements, Stereo eyetracking, Head movement

## Abstract

Traditional video-based eyetrackers require participants to perform an individual calibration procedure, which involves the fixation of multiple points on a screen. However, certain participants (e.g., people with oculomotor and/or visual problems or infants) are unable to perform this task reliably. Previous work has shown that with two cameras one can estimate the orientation of the eyes’ optical axis directly. Consequently, only one calibration point is needed to determine the deviation between an eye’s optical and visual axes. We developed a stereo eyetracker with two USB 3.0 cameras and two infrared light sources that can track both eyes at ~ 350 Hz for eccentricities of up to 20°. A user interface allows for online monitoring and threshold adjustments of the pupil and corneal reflections. We validated this tracker by collecting eye movement data from nine healthy participants and compared these data to eye movement records obtained simultaneously with an established eyetracking system (EyeLink 1000 Plus). The results demonstrated that the two-dimensional accuracy of our portable system is better than 1°, allowing for at least ± 5-cm head motion. Its resolution is better than 0.2° (*SD*), and its sample-to-sample noise is less than 0.05° (RMS). We concluded that our stereo eyetracker is a valid instrument, especially in settings in which individual calibration is challenging.

**Electronic supplementary material:**

The online version of this article (10.3758/s13428-018-1026-7) contains supplementary material, which is available to authorized users.

Standard video-based eyetrackers rely on an individual calibration procedure in which participants are asked to fixate multiple small targets at known locations. This calibration is necessary to convert the image features of the eyes into estimates of the point of gaze (POG) on the screen (Hansen & Ji, 2010). Most healthy adults are able to perform such a calibration procedure effortlessly. However, if participants are unable to reliably fixate small stimuli—for example, in the case of oculomotor deficits, low vision, or reduced attention—accurate calibration is not possible. As a result, no reliable gaze data can be obtained with standard video-based eyetrackers in these participants.

The scleral search coil method can be an alternative for some of these participants. This technique measures electromagnetic induction in a copper coil that is embedded in a contact lens (Collewijn, van der Mark, & Jansen, 1975; Robinson, 1963). Each search coil can be accurately calibrated before use, so that only one in vivo calibration point is needed to determine the orientation of the coil with respect to the visual axis of the participant’s eye (Leigh & Zee, 2015). This method has very high temporal and spatial resolution, allowing even for smaller eye movements to be studied. However, a major disadvantage of this method is its somewhat invasive and uncomfortable nature. The cornea needs to be anesthetized, and the recording time is typically limited to about 30–45 min (but see also Sprenger et al., 2008). These disadvantages make it an unsuitable technique for most clinical settings and for eye movement recordings in (young) children (Van Der Geest & Frens, 2002). Therefore, researchers have developed noninvasive video-based eyetracking techniques with two cameras that rely on a simplified calibration procedure (Chen, Tong, Gray, & Ji, 2008; Guestrin & Eizenman, 2006; Kohlbecher et al., 2008; Lai, Shih, & Hung, 2015; Nagamatsu, Kamahara, Iko, & Tanaka, 2008; Shih, Wu, & Liu, 2000; Zhu & Ji, 2007). With these stereo eyetracking methods, one can estimate the position of the eye and the orientation of its optical axis from the stereo images and the known geometry of the setup. Only the deviation between the optical and visual axes needs to be determined, which can be done through a one-point calibration procedure. Note that even without this in vivo calibration, the changes in orientation of the optical axis still accurately reflect changes in eye orientation; they only display a fixed offset with respect to the gaze angles. The spatial accuracy of the proposed prototypes of stereo eyetrackers is typically around 1 deg (Chen et al., 2008; Lai et al., 2015; Zhu & Ji, 2007), or even below 1 deg (Guestrin & Eizenman, 2011; Shih & Liu, 2004). This is sufficient for a range of eyetracking applications. However, the sampling rates of those systems range between 20 and 30 Hz (Chen et al., 2008; Guestrin & Eizenman, 2011; Lai et al., 2015; Shih & Liu, 2004; Zhu & Ji, 2007), which is not enough to analyze the kinematics of rapid eye movements (Mack, Belfanti, & Schwarz, 2017). Since the temporal resolution of eyetrackers affects their estimates of saccade peak velocities and other kinematic parameters, it has been recommended to use sampling rates of at least 200 Hz (Inchingolo & Spanio, 1985), 250 Hz (Mack et al., 2017; Schmitt, Muser, Lanz, Walz, & Schwarz, 2007), or even 300 Hz (Juhola, 1986). Therefore, our aim was to develop a stereo eyetracker with a sampling rate of at least 250 Hz.

The accuracy of eyetrackers is typically evaluated by measuring the deviation between the location of the visual target and the reconstructed point of gaze (Holmqvist et al., 2011). However, it is known that participants do not always look exactly at the center of the target (Thaler, Schütz, Goodale, & Gegenfurtner, 2013). In addition, the individual characteristics of participants, such as iris color and the physiology and anatomy of the eye can lower the accuracy of video-based eyetrackers (Holmqvist et al., 2011). Therefore, the second aim of the present study was to validate the accuracy of our stereoscopic eyetracker against the accuracy of an established high-speed remote eyetracking system, the EyeLink 1000 Plus, by recording simultaneously with the two systems.

Previous studies have shown that the accuracy of standard remote eyetrackers deteriorates if the head moves (Hessels, Cornelissen, Kemner, & Hooge, 2014; Niehorster, Cornelissen, Holmqvist, Hooge, & Hessels, 2017). In addition, recent simulations have revealed that head movements could also reduce the accuracy of stereo eyetrackers (Barsingerhorn, Boonstra, & Goossens, 2017). Therefore, we assessed the robustness against head movements of the two systems by testing the accuracy of the EyeLink 1000 Plus and the stereoscopic eyetracker for nine different head positions.

## Method

### Stereo eyetracker hardware

**Fig. 1:**
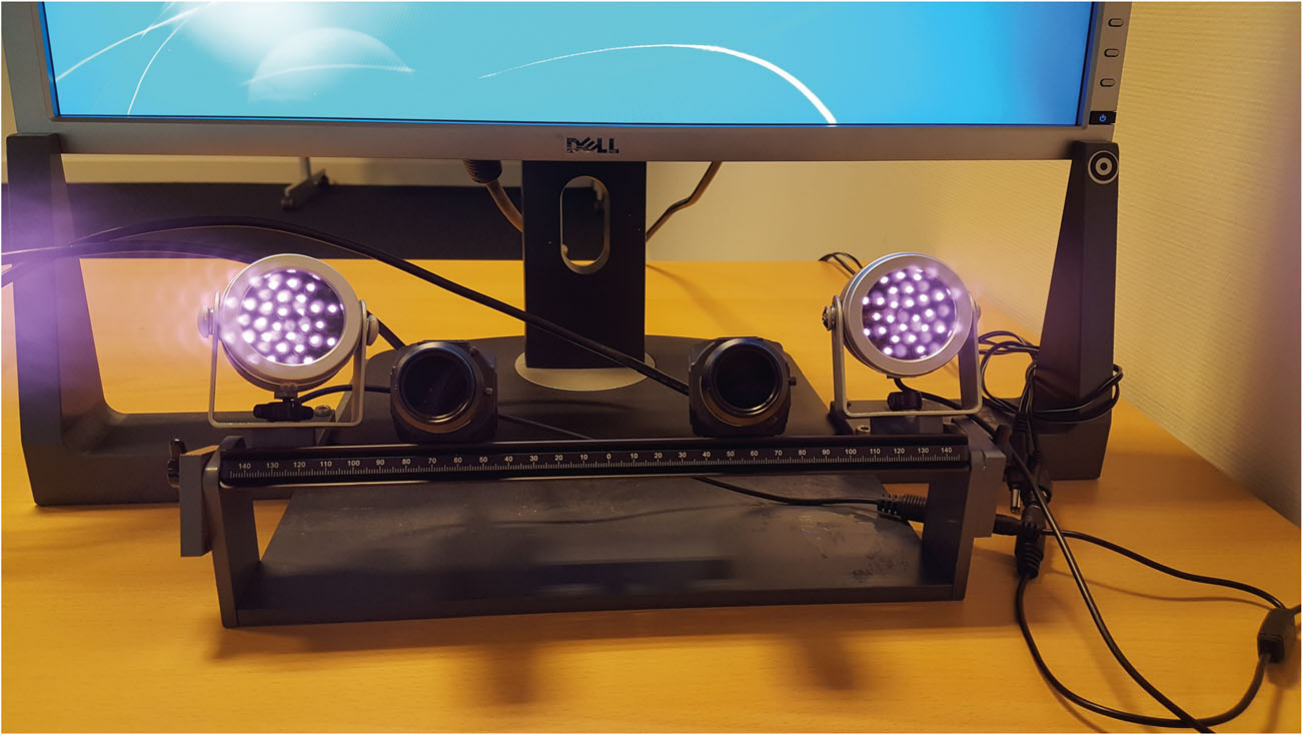
The stereo eyetracking system. The hardware consists of two infrared illuminators and two USB 3.0 cameras connected to a laptop computer

The stereo eyetracker is shown in Fig. 1. It consisted of two USB 3.0 cameras (Lumenera lt225 NIR, Lumenera Corp., Ottawa, Canada, pixel size 5.5 × 5.5 *μ*m) and two 850-nm infrared illuminators (Abus TV6700, ABUS KG, Wetter, Germany) mounted on an optic rail. The cameras were positioned ~ 12 cm apart, with their optical axes directed toward the location of the participants’ eyes. The first illuminator was placed ~ 6 cm to the right of Camera 1 (the right one in that figure), and the second illuminator was placed ~ 6 cm to the left of Camera 2.

The temporal resolution of the cameras depended on their spatial resolution settings, due to the limited amount of data that can be transferred over a USB 3.0 connection. At full spatial resolution (2,044 × 1,088 pixels), the cameras could film at ~ 180 fps, whereas the cameras reached ~ 380 fps at 1,048 × 480 pixels. For the present eyetracking application, we selected the latter option. The lenses with manual focus and diaphragm had a focal length of 16 mm (Navitar NMV-16M23, Navitar Inc, Rochester, NY, USA). Infrared-passing filters (UV/Vis-Cut R-72; Edmund Optics Inc., Barrington, NJ, USA) that passed wavelengths > 720 nm were added on the lenses to block light in the visible spectrum. The eyetracking software was executed on a laptop (Dell M3800; Dell Inc., Round Rock, TX, USA) equipped with eight 2.3-Ghz central processing units (Intel core i7-472HQ, Santa Clara, CA, USA), an OpenGL graphics card (Nvidia Quadro K1100M; Santa Clara, CA, USA), and a 64-bit Windows 7 Professional operating system (Service Pack 1, Microsoft Corp., Redmond, WA, USA). Each camera was connected to a separate USB 3.0 bus to achieve high camera frame rates.

### System calibration

The system was calibrated in two steps. First, the internal and external camera parameters were obtained through a stereo camera calibration procedure in which images of a calibrated checkerboard pattern were taken while it was moved around the cameras at various angles (Matlab computer vision toolbox, Matlab R2016b, MathWorks, Inc., Natick, MA, USA). This calibration procedure allowed us to correct for lens distortions (using the function undistortPoints from the MATLAB computer vision toolbox) and express the image coordinates in both cameras in a common world-centered frame of reference using a right-handed Cartesian coordinate system with its origin located at the nodal point of Camera 1. The *x*- and *y*-axis of the tracker’s coordinate system were parallel to the image plane of Camera 1, and the positive *z*-axis pointed away from it (i.e., toward the participant). In the second step, the position of the illuminators and the position and orientation of the screen were determined. The illuminators and the screen were not directly observed by the cameras, as they were located behind the camera system. Therefore, a planar mirror with a dot stimulus pattern attached to its surface was used to observe the virtual images of the illuminators and the screen, as suggested in several studies (Beymer & Flickner, 2003; Chen et al., 2008; Shih & Liu, 2004; Zhu & Ji, 2007). To reconstruct the position and orientation of the mirror, we determined the 3-D location of the markers from their image coordinates in each of the two cameras by means of triangulation. Both cameras also observed virtual images of the illuminators and the computer screen in the mirror. Thus, also the 3-D locations of the virtual images of the illuminators and the screen could be obtained through triangulation. The 3-D positions of the illuminators were then determined from the 3-D locations of their virtual images behind the mirror and the 3-D position and orientation of the mirror itself. In a similar way the 3-D position and orientation of the screen were determined from the virtual image of a dot stimulus pattern that was displayed on the screen during the procedure. We performed all triangulations with the function triangulate from the computer vision toolbox of Matlab, as it automatically converts the image coordinates into world-centered system coordinates using the parameters from the stereo camera calibration.

### Stereo eyetracker software

We developed the eyetracking software in Visual Studio 2012 with C# as the programming language. The LuCam SDK V6.3 (Lumenera Corporation, Ottawa, Canada) camera driver was used to set the acquisition parameters (shutter time, resolution, etc.) and capture the images from the two cameras. The EmguCV library (OpenCV [Bradski & Kaehler, 2008] wrappers for C#) was used for online image processing. The stereo eyetracking software, as well as the offline gaze reconstruction algorithms described below, are available at https://github.com/Donders-Institute/Stereo-gaze-tracking.

**Fig. 2:**
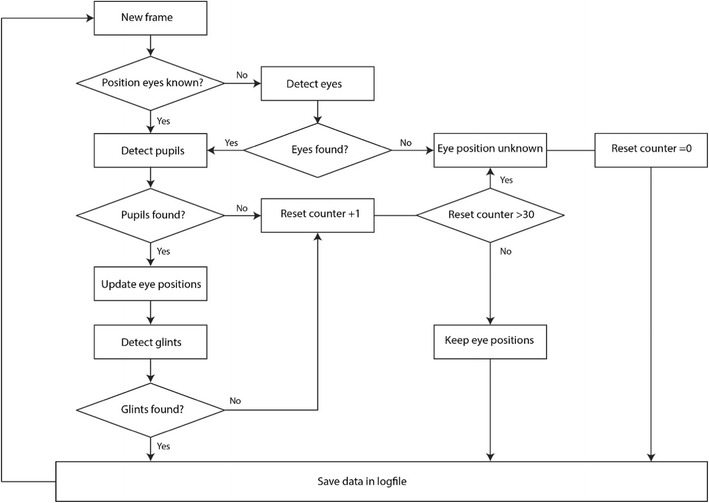
Flow chart of the image processing for each camera. First the eyes are detected, after which the pixel coordinates of the pupil and glints are extracted. A reset counter is used to force the software to redetect the eyes only if no pupil or glints are detected in 30 consecutive frames. If only one eye is found, the software continues tracking that eye for 30 frames. After 30 frames, the eyes are redetected in order to continue binocular tracking

The eyetracking program supports tracking the pupil and corneal reflections (glints) for both eyes with two cameras. It can run simultaneously with (custom) stimulus presentation software on the same computer, which eliminates the need for a separate eyetracking computer. The code for detecting, segmenting and tracking the pupil and glints was adapted from the open source software of the ITU Gaze Tracker (San Agustin et al., 2010) which was designed for single-camera setups. The acquisition and analysis of the images from the two cameras run in separate threads. A simplified flow chart of the processing within each thread is shown in Fig. 2. First, the eyes are detected by a Haar cascade classifier (Viola & Jones, 2001), which is part of the EmguCV library. Once the eyes are found, the pixel coordinates of the pupil centers and the glints are estimated. To extract the pupil, the image of the eye is segmented by thresholding the image. The center of the pupil is then estimated at subpixel resolution by fitting an ellipse (least squares method) to the pixel coordinates of the pupil contour. Subsequently, the glints are detected with a different intensity threshold. The centroids of the glints provide an estimation of the center of the glints at subpixel resolution. The center of the pupil is used to update the eye positions to allow for robust tracking in the presence of head movements. If the software fails to detect a pupil or glints, the eye positions might have changed due to head movements, or the participant could have blinked. Here, we had to balance between robustness and speed of the eyetracking. Detecting the eyes is relatively slow (~ 16 ms) because of the computational load. Therefore, redetecting the eyes too quickly after a failure to detect a pupil or glints is inefficient. The participant could have blinked, in which case the tracking could continue immediately after the blink without redetection necessary. However, if the software waits too long to redetect the eyes, data might be lost if the eyes truly shifted due to head movements. Therefore, the software redetects the eyes only if no pupil or glints are found in 30 consecutive frames. The timestamps and the pixel coordinates of the pupils and glints are saved in a data file.

**Fig. 3:**
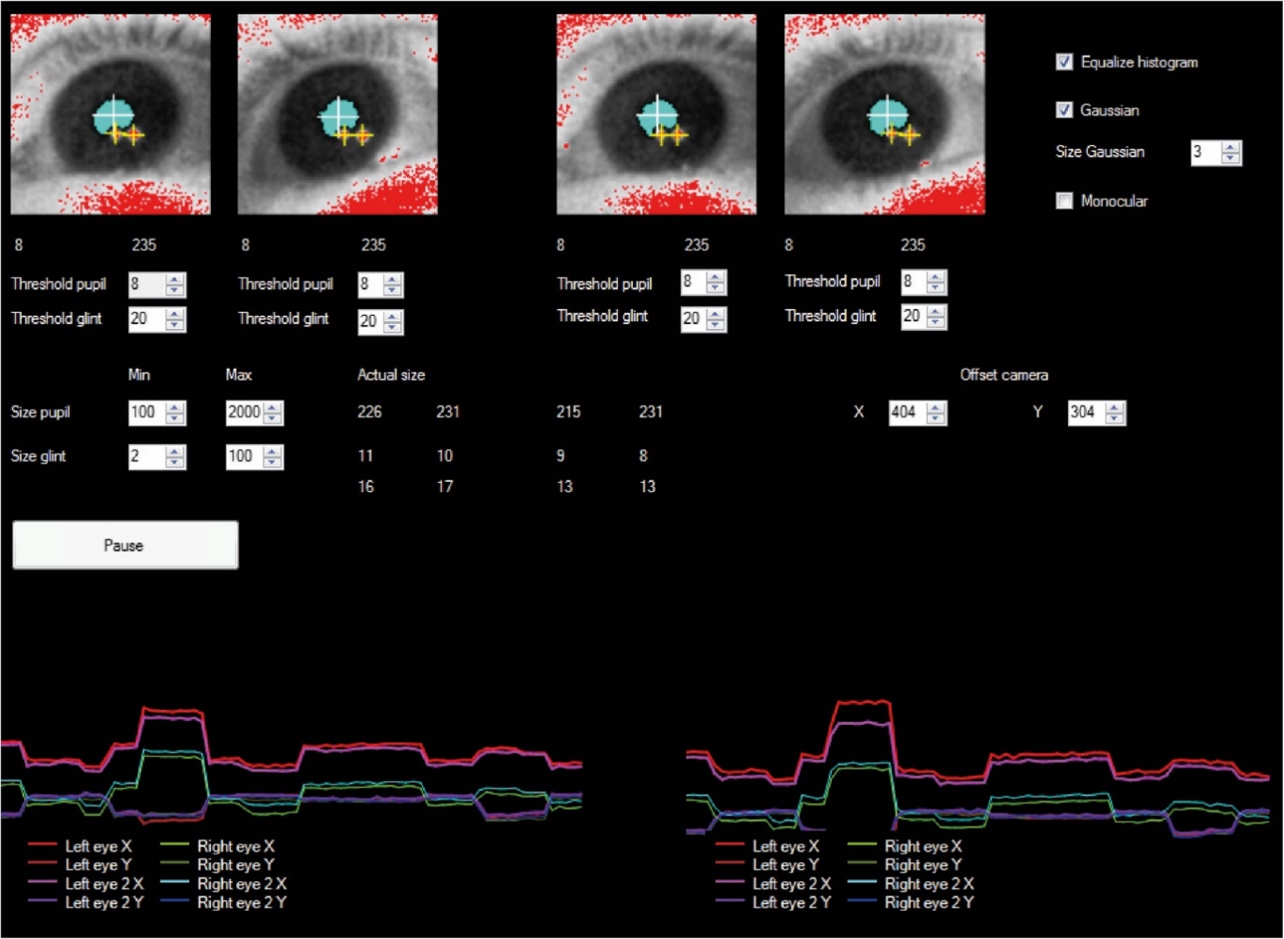
User interface of the stereo eyetracker. The online image segmentation is visible in the four eye images. The two images on the left are for the left and right eyes of the participant as observed by the left camera. The two images on the right are the participant’s left and right eyes as observed by the right camera. The image segmentations of the pupil (blue) and glints (red) are shown, and the centers of the pupil and glints are indicated with crosshairs. The thresholds for the image segmentation and the size limits of the pupils and glints can be adjusted. Online feedback is provided through the pupil–glint vectors, in the lower panels

Figure 3 shows a screenshot of the online display. Because both eyes are filmed by two cameras, four eye images are visible. The results of image thresholding are shown in blue, for the pupil detection, and in red, for the glint detection. The estimated centers of the pupils are shown with white crosshairs. The estimated centers of the glints are shown with yellow crosshairs. The thresholds for the image segmentation can be adjusted separately for each eye in each camera, whereas the upper and lower bounds for pupil size and glint size are set to common values. For most participants (~ 2/3), the default values shown in Fig. 3 yield accurate results. Storage of the tracking data can be paused, in order to prevent unnecessarily large data files. In addition, online feedback is given by showing the horizontal and vertical components of the pupil–glint vectors as a function of time for both cameras over a period of ~ 3 s. The pupil–glint vectors are used in most standard eyetrackers to calculate the point of gaze by using the mapping obtained through the calibration procedure (Hansen & Ji, 2010). In our online display it does not reflect the exact point of gaze or the exact gaze direction, but it gives a clear indication of the quality and noise level of the data. For example, the occurrence of a number of larger and smaller saccades is clearly visible in these uncalibrated signals.

In addition, a histogram equalizer (EmguCV) can be used to improve the image segmentation, and a Gaussian filter can be applied to reduce the effect of pixel noise on the estimation of the pixel coordinates of the pupils and glints. For some applications, monocular tracking may be preferred. Therefore, the stereo tracker can be set in a monocular tracking modus. Furthermore, it is possible to adjust which part of the camera sensor is used, to make sure the participant is well within the selected field of view.

We have included two videos of the online display in Supplement 1 and 2 to illustrate the tracking stability of the system. Both participants (the first and last authors) were head-free and looked at the online display window, which was placed on the stimulus screen for illustration purposes. The images that are visible on the laptop screen are the unprocessed images from the two cameras.

### Gaze reconstruction

The reconstruction of gaze was performed offline in Matlab R2016b (MathWorks, Inc., Natick, MA, USA). Multiple researchers have demonstrated that with two cameras and two light sources at known locations, the 3-D location of the eye as well as the 2-D orientation of the optical axis of the eye can be approximated by estimating the center of the virtual pupil (**p**_**v**_**)** and the center of corneal curvature (**c**) (Chen et al., 2008; Guestrin & Eizenman, 2006; Shih & Liu, 2004; Zhu & Ji, 2007). Because image acquisition in the two cameras ran asynchronously, for both cameras the pixel coordinates of the pupils and glints were first interpolated at the timestamps at which the other camera had captured an image of the eyes.

**Fig. 4:**
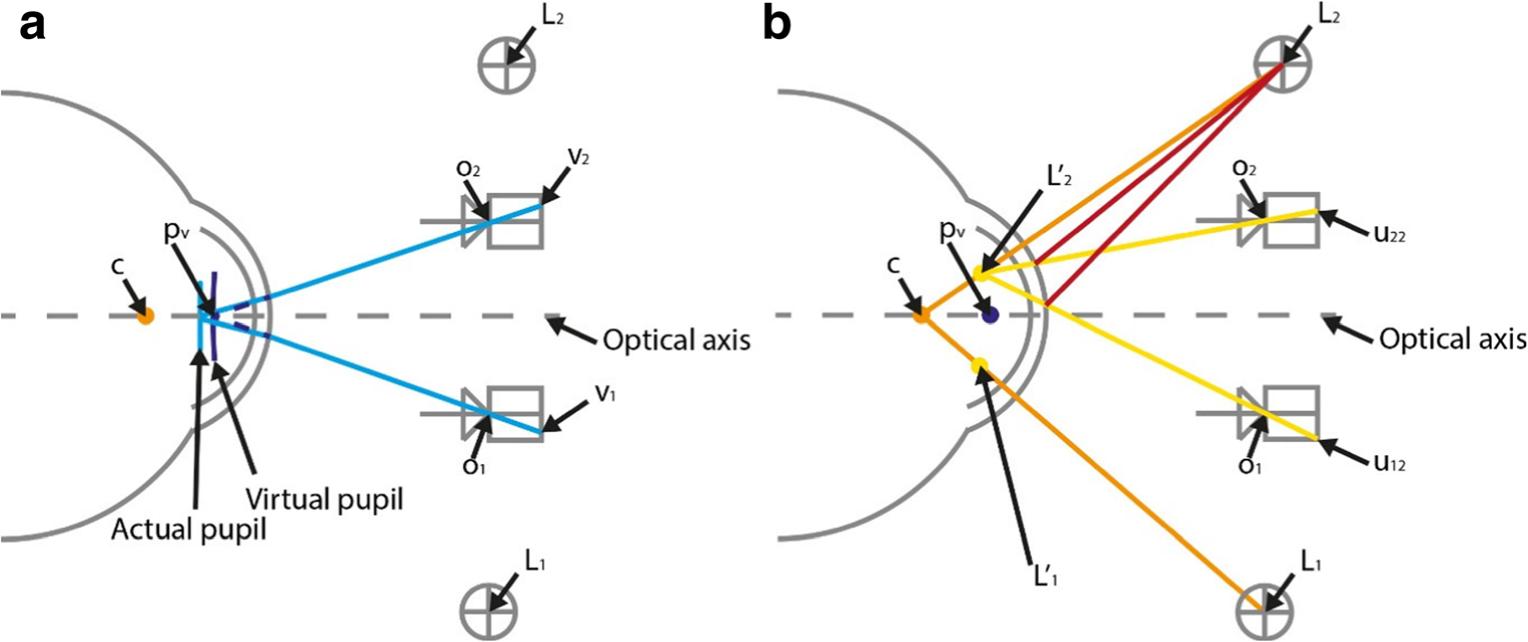
Ray-tracing diagrams (not to scale), showing a schematic top view of the two cameras, the two infrared illuminators, and one eye. (A) The center of the pupil projections onto each camera (v_1_ and v_2_) and the nodal points of those cameras (o_1_ and o_2_) were used to triangulate the center of the virtual pupil p_*v*_. (B) For each illuminator L_*j*_, causing glint u_1*j*_ in Camera 1 and glint u_2*j*_ in Camera 2, its virtual image L^′^_*j*_ was obtained through triangulation (yellow lines). Subsequently, the center of corneal curvature c was estimated by intersecting the line through illuminator L_1_ and its virtual image L^′^_1_ with the line through illuminator L_2_ and its virtual image L^′^_2_

Due to the refraction of light rays at the corneal surface, the cameras do not observe the actual pupil (Fig. 4A). They see only a virtual image of the pupil. Different approaches have been used to estimate the center of the virtual pupil. Some of these methods correct for refraction at the corneal surface (Lai et al., 2015; Nagamatsu et al., 2008) by assuming a spherical shape of the cornea, whereas others do not correct for the refraction (Chen et al., 2008; Guestrin & Eizenman, 2006; Zhu & Ji, 2007). We decided not to correct for refraction at the corneal surface, because it has been shown that, even if a more realistic model of the cornea is used, the location of the center of the virtual pupil remains within 0.2 deg of the optical axis (Barsingerhorn et al., 2017). Therefore, the center of the virtual pupil was triangulated from the image points ***v***_**1**_ and ***v***_**2**_ of the virtual pupil in the two cameras and the nodal points ***o***_**1**_ and ***o***_**2**_ of the cameras (Fig. 4A) by computing ***p***_**v**_ from the least squares solution of

1 $$ \left\{\begin{array}{c}{\boldsymbol{p}}_{\boldsymbol{v}}={\boldsymbol{o}}_{\mathbf{1}}+{\lambda}_1\left({\boldsymbol{v}}_{\mathbf{1}}-{\boldsymbol{o}}_{\mathbf{1}}\right)\\ {}{\boldsymbol{p}}_{\boldsymbol{v}}={\boldsymbol{o}}_{\mathbf{2}}+{\lambda}_2\left({\boldsymbol{v}}_{\mathbf{2}}-{\boldsymbol{o}}_{\mathbf{2}}\right)\end{array}\right. $$

Different approaches have been used to obtain the center of corneal curvature (Chen et al., 2008; Guestrin & Eizenman, 2006; Shih & Liu, 2004; Zhu & Ji, 2007), all of which assume that the cornea acts as a spherical mirror during the process of glint formation. We used the approach suggested by Zhu and Ji. Based on the reflection law of convex mirrors, this method assumes that each camera observes a virtual image of the IR illuminator at the same location regardless of the camera’s location. The position of the virtual image of the illuminator is then only determined by the actual position of the illuminator and by the location of the eye. With two cameras the 3-D location of the virtual image of an illuminator can be triangulated from the corresponding glint coordinates in the two cameras (Fig. 4B). That is, for each illuminator ***L***_**j**_, causing glint ***u***_**1j**_ in Camera 1 and glint ***u***_**2j**_ in Camera 2, its virtual image **L’**_**j**_ was obtained through:

2 $$ \left\{\begin{array}{c}\boldsymbol{L}{\prime}_{\boldsymbol{j}}={\boldsymbol{o}}_{\mathbf{1}}+{\lambda}_1\left({\boldsymbol{u}}_{\mathbf{1}\boldsymbol{j}}-{\boldsymbol{o}}_{\mathbf{1}}\right)\ \\ {}\boldsymbol{L}{\prime}_{\boldsymbol{j}}={\boldsymbol{o}}_{\mathbf{2}}+{\lambda}_2\left({\boldsymbol{u}}_{\mathbf{2}\boldsymbol{j}}-{\boldsymbol{o}}_{\mathbf{2}}\right)\ \end{array}\right. $$

Subsequently, the center of corneal curvature ***c*** was estimated by intersecting the line through illuminator ***L***_**1**_ and its virtual image ***L***^′^_**1**_ with the line through illuminator ***L***_**2**_ and its virtual image ***L***^′^_**2**_:

3 $$ \left\{\begin{array}{c}\boldsymbol{c}={\boldsymbol{L}}_{\mathbf{1}}+{\lambda}_1\left({\boldsymbol{L}}_{\mathbf{1}}-\boldsymbol{L}{\prime}_{\mathbf{1}}\right)\ \\ {}\boldsymbol{c}={\boldsymbol{L}}_{\mathbf{2}}+{\lambda}_2\left({\boldsymbol{L}}_{\mathbf{2}}-\boldsymbol{L}{\prime}_{\mathbf{2}}\right)\ \end{array}\right. $$

In practice, the 3-D reconstructions using triangulation are not perfect. Due to image noise and an aspherical cornea (Barsingerhorn et al., 2017; Chen et al., 2008; Guestrin & Eizenman, 2011), often the lines do not intersect. Therefore, the position at which the distance between the lines is minimal is taken as the 3-D position. This introduces errors in the estimation of ***c*** and ***p***_**v**_. Chen et al. proposed a method to reduce the variability resulting from image noise by assuming that for a given participant the distance *K* between ***c*** and ***p***_**v**_ remains constant. Because image noise mainly affects the localization along the *z*-axis of the tracker’s coordinate system (i.e., in the cameras viewing direction), the *x* and *y* coordinates of the virtual pupil are kept fixed, whereas its *z*-coordinate is changed to satisfy the constraint that *K* is fixed. Given the estimated center of corneal curvature ***c*** = (*x*_*c*_, *y*_*c*_, *z*_*c*_) and the virtual pupil center ***p***_***v***_ = (*x*_*pv*_, *y*_*pv*_, *z*_*pv*_), the *z*-coordinate of ${\boldsymbol{p}}_{\boldsymbol{v}}^{\prime }$is computed as:

4 $$ z{\prime}_{pv}={z}_c-\sqrt{K^2-{\left({x}_c-{x}_{pv}\right)}^2-{\left({y}_c-{y}_{pv}\right)}^2}, $$

where *K* is a participant/eye-specific value.

In the next step, the *x*-, *y*-, and *z*-coordinates of ${\boldsymbol{p}}_{\boldsymbol{v}}^{\prime }$and **c** in the tracker’s coordinate system were mapped onto *x*-, *y*-, and *z*-coordinates of a right-handed Cartesian coordinate system whose *XY*-plane was coincident with the orientation of the screen and with its origin located at the center of the screen. In this stimulus coordinate system the *x*-axis was horizontal, the *y*-axis vertical, and the positive *z*-axis came out of the screen (i.e., toward the participant). This coordinate transformation involved translations, ***T***, and rotations, ***R***, determined from the system calibration procedure (see above):

5 $$ {\displaystyle \begin{array}{c}{\boldsymbol{P}}_{\boldsymbol{v}}^{\prime }=\boldsymbol{T}\left(\boldsymbol{R}\ {\boldsymbol{p}}_{\boldsymbol{v}}^{\prime}\right),\\ {}\boldsymbol{C}=\boldsymbol{T}\left(\boldsymbol{R}\ \boldsymbol{c}\right)\end{array}} $$

Following Guestrin and Eizenman (2006), the orientation of the optical axis of the eye was then described by the horizontal (pan) angle *θ*_eye_ and the vertical (tilt) angle *ϕ*_eye_ where the origin of the coordinate system is translated to the center of corneal curvature. The angles *θ*_eye_ and *ϕ*_eye_ were obtained from ***C*** and ${\boldsymbol{P}}_{\boldsymbol{v}}^{\prime }$as follows:

6 $$ \frac{{\boldsymbol{P}}_{\boldsymbol{v}}^{\prime }-\boldsymbol{C}}{\left\Vert {\boldsymbol{P}}_{\boldsymbol{v}}^{\prime }-\boldsymbol{C}\right\Vert }=\left[\begin{array}{c}\cos {\phi}_{\mathrm{eye}}\sin {\theta}_{\mathrm{eye}}\\ {}\sin {\phi}_{\mathrm{eye}}\\ {}-\cos {\phi}_{\mathrm{eye}}\cos {\theta}_{\mathrm{eye}}\end{array}\right], $$

The orientation of the visual axis, which defines the direction of gaze, was then estimated from the orientation of the optical axis and the deviation between the optical axis and visual axis using

7 $$ \hat{\boldsymbol{g}}=\left[\begin{array}{c}\cos \left({\phi}_{\mathrm{eye}}+{\beta}_{\mathrm{eye}}\right)\sin \left({\theta}_{\mathrm{eye}}+{\alpha}_{\mathrm{eye}}\right)\\ {}\sin \left({\phi}_{\mathrm{eye}}+{\beta}_{\mathrm{eye}}\right)\\ {}-\cos \left({\phi}_{\mathrm{eye}}+{\beta}_{\mathrm{eye}}\right)\cos \left({\theta}_{\mathrm{eye}}+{\alpha}_{\mathrm{eye}}\right)\end{array}\right], $$

where *α*_eye_ and *β*_eye_ are the horizontal and vertical angles between the visual axis and the optical axis, respectively. These angles *α*_eye_ and *β*_eye_ were estimated from the horizontal and vertical angles of the optical axis with respect to the line of sight during a single point calibration procedure that involved fixation of a small target at the center of the screen (see below). The median length of the ***c − p***_***v***_ vector measured during this procedure provided the participant-specific value of *K* used in Eq. 4.

Since the visual axis goes through the center of corneal curvature ***C***, the point of gaze (POG) on the screen can be estimated from the following parametric equation (Guestrin & Eizenman, 2006):

8 $$ \boldsymbol{POG}=\boldsymbol{C}+{\lambda}_g\widehat{\boldsymbol{g}}, $$

where *λ*_*g*_ is the distance from ***C*** at which the line of sight intersects the screen. Given the estimated center of corneal curvature ***C*** **=** (*X*_*c*_, *Y*_*c*_, *Z*_*c*_), and because the screen is a planar scene at *Z* = 0, the value of *λ*_*g*_ is given by

9 $$ {\lambda}_g=-\frac{Z_c}{-\cos \left({\phi}_{e\mathrm{ye}}+{\beta}_{\mathrm{eye}}\right)\cos \left({\theta}_{\mathrm{eye}}+{\alpha}_{\mathrm{eye}}\right)}. $$

This reconstruction of ***POG*** does not account for the kinematics of the eyeball (but see Guestrin & Eizenman, 2010; Nagamatsu et al., 2008, for an alternative procedure that incorporates Listing’s law under the assumption that Listing’s plane is parallel to the *XY*-plane).

The correction for the effect of image noise on the *z*-component of the 3-D reconstructions (Eq. 4) worked well to reduce the noise level up to ~ 50%. However, it turned out that it introduced systematic errors in the accuracy of the stereo tracker by systematically under- or overestimating the gaze angles up to ~ 15% if the actual length of the ***c − p***_***v***_ vector differed substantially from *K*. This is in line with recent simulations of stereo eyetracking methods, which indicated that the length of the ***c − p***_***v***_ vector varies systematically as a function of head position, pupil size and gaze angles due to asphericity of the cornea (Barsingerhorn et al., 2017). We first attempted to account for these variations in the length the ***c − p***_***v***_ vector by replacing *K* in Eq. 4 with a low-pass filtered measure of the actual length of the ***c − p***_***v***_ vector, *K*_actual_. Although this approach did reduce the systematic errors to some extent, it could not adequately account for translations of the head. From this we inferred that the systematic under- or overestimation of the gaze angles is not only due to variations in length of the ***c − p***_***v***_ vector. Indeed, the consequences of the aspherical properties of the cornea are complex. Simulations with an aspherical model of the cornea suggest that the systematic variation in length of the ***c − p***_***v***_ vector is caused by shifts of the virtual pupil and a mislocalization of the “center of corneal curvature” (through triangulation or other proposed methods) because an aspherical cornea does not have a unique center of curvature (Barsingerhorn et al., 2017). The consequences of shifts of the virtual pupil are probably small, because they are primarily along the optical axis of the eye, but mislocalization of the “center of corneal curvature” introduces more severe errors, because this can put the estimate of ***c*** off the optical axis in a way that varies systematically with the position and orientation of the eye with respect to the cameras. From this, we inferred that corrections for systematic errors in the reconstruction of the POG would have to include the 3-D position of the eye, as well. Empirically, we found that the reconstruction errors of the POG can be attenuated significantly with a variable gain factor that reflects the difference between *K* and the actual length of the ***c − p***_***v***_ vector and that acts on both the eye position term and the gaze orientation term of Eq. 8 in the following way:

10 $$ \boldsymbol{PO}{\boldsymbol{G}}_{\mathbf{corrected}}=\frac{K}{K_{\mathrm{actual}}}\left(\boldsymbol{C}+{\lambda}_g\widehat{\boldsymbol{g}}\right) $$

where *K*_actual_ is a filtered measure of the actual length of the ***c − p***_***v***_ vector. A median filter with a width of 20 samples (using the function medfilt1, Matlab 2016b) was used. From the corrected POG, we obtained the corrected direction of gaze,

11 $$ {\widehat{\boldsymbol{g}}}_{\mathbf{corrected}}=\frac{POG_{\mathrm{corrected}}-\boldsymbol{C}}{\left\Vert {POG}_{\mathrm{corrected}}-\boldsymbol{C}\right\Vert }, $$

and subsequently, the corrected gaze angles *θ*_*eye*corr_ and *ϕ*_*eye*corr_ were obtained through the formula

12 $$ {\hat{\boldsymbol{g}}}_{\mathbf{corrected}}=\left[\begin{array}{c}\cos \kern0.10em {\phi}_{eye\mathrm{corrected}}\kern0.10em \sin \kern0.10em {\theta}_{eye\mathrm{corrected}}\\ {}\sin \kern0.10em {\phi}_{eye\mathrm{corrected}}\\ {}-\cos \kern0.10em {\phi}_{eye\mathrm{corrected}}\kern0.10em \cos \kern0.10em {\theta}_{eye\mathrm{corrected}}\end{array}\right] $$

The proposed corrections also worked when the head was translated (see the Results below).

Note that Eqs. 8–11 perform the corrections in stimulus coordinates. It may be possible to apply the correction in the tracker’s coordinate system. However, because *K*_actual_ varies as a function of head translations, and because *K* is estimated from a one-point calibration in which the participant looks at the center of the screen (see below), we think it is essential to use this reference point as the origin for the corrections. Otherwise the correction could introduce additional errors, especially if the position of the eye changes due to head motion.

### Filtering

The coordinates of ***P***′_***v***_ and ***C*** in the stimulus coordinate system were filtered with a median filter with a width of 20 samples (using the function medfilt1, Matlab 2016b), the same filter that was applied to *K*_actual_. We decided to use a median filter, because this type of nonlinear digital filter smoothes signals by attenuating noise whilst preserving edges in signals. For moderate to small levels of noise they prove to be efficient in removing small noise peaks with almost no impact on the dynamics of saccades (Juhola, 1991)*.*

### Eyetracker validation study

**Fig. 5:**
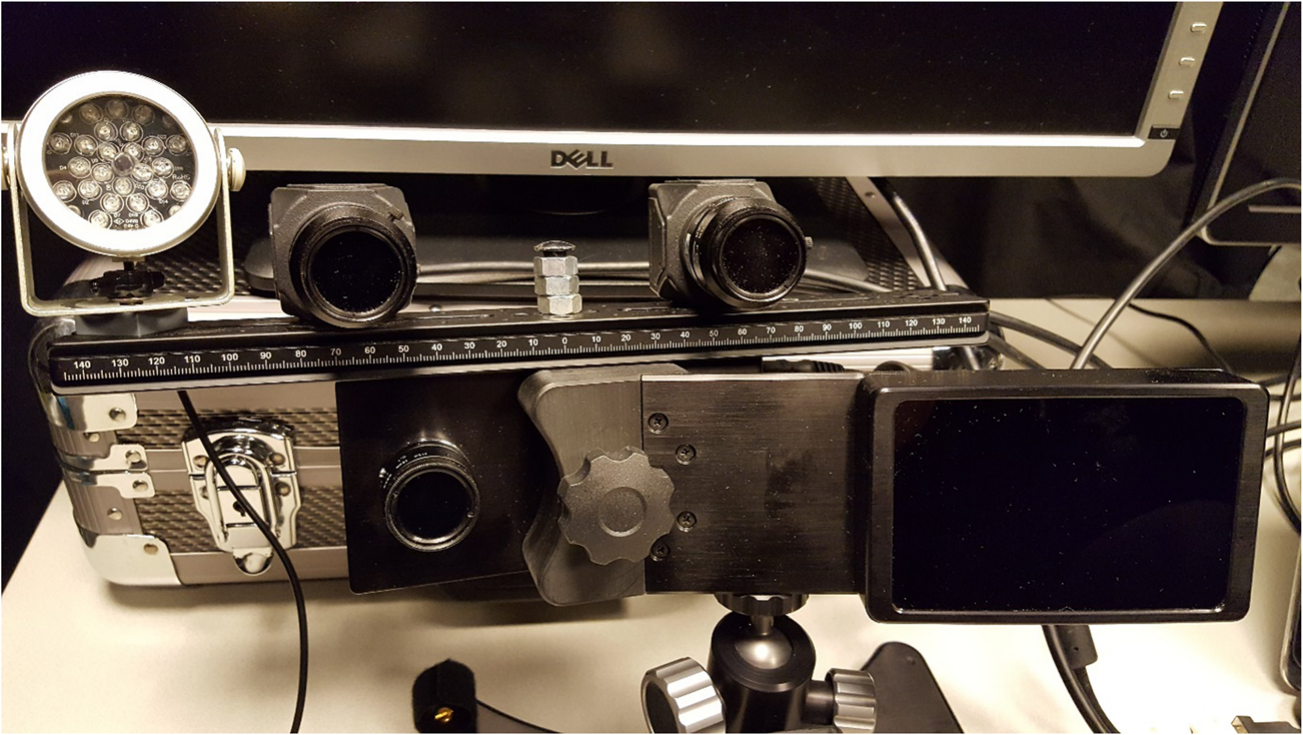
Experimental setup for the validation study. The stereo tracker was mounted on top of the EyeLink camera

To validate the stereo eyetracker, we performed a study in which we recorded eye movements simultaneously with the stereo tracker and an EyeLink 1000 Plus in remote tracking mode (which relies on tracking the pupil–glint vector). Because the EyeLink also has an infrared illuminator (890 nm), we removed one of the infrared illuminators of the stereo eyetracker and placed the stereo tracker on top of the EyeLink (Fig. 5). The EyeLink camera (2,048 × 2,048 pixels) was equipped with a 16-mm C-mount lens supplied by the manufacturer and was placed underneath the stimulus screen using its desktop mount. The camera screw was aligned with the center of the monitor, and the top of the illuminator was approximately at the height of, and parallel with, the lower edge of the monitor. The eye-to-camera distance was ~ 55 cm, which is within the recommended range (between 40 and 70 cm). The camera-to-screen distance was ~ 10 cm. EyeLink data were recorded at 500 Hz, with the sample filtering level set to “Standard.” This is a heuristic filter with a width of three samples (Stampe, 1993). Nine healthy participants (25 ± 4 years) with normal visual acuity were included in the validation study. None of the participants wore glasses or contact lenses. All participants gave informed consent before the start of the experiment. A bite-board was used to stabilize the head ~ 65 cm from the screen. The participants performed a visually guided saccade task at nine different head positions. The participants started in the central head position, in which they were positioned in front of the center of the screen by placing their “cyclopean eye” on the *z*-axis of the stimulus coordinate system at a distance of 65 cm. In the other conditions the bite-board was translated ± 5 cm horizontally and ± 3 cm vertically from the central position.

Before the start of the experiment, a small target sticker was placed on the participant’s forehead, just above the eyebrows, and a monocular calibration procedure was performed at the central head position. During calibration of the right eye, the left eye was occluded, and during calibration of the left eye the right eye was occluded. This forced the participants to fixate the calibration targets with the eye being calibrated. We made sure that the EyeLink software did not confuse the glints caused by its own infrared illuminator and the (significantly) weaker one from the infrared illuminator of the stereo tracker by raising the glint thresholds to sufficiently high levels (without losing the glints while the participant looked at the edges of the stimulus display). For the EyeLink we performed a 13-point calibration procedure, after which we performed a 13-point validation to check the quality of the calibration. The calibration was accepted only if the EyeLink software indicated that the quality was good, otherwise the calibration was repeated. For the stereo tracker we used a one-point calibration. Fixation of the central target during the EyeLink calibration was used to estimate the deviation between the optical and visual axes of the eye and the participant-specific value of *K*. No recalibration was performed at the other head positions. In addition, we did not apply any drift corrections.

In the experimental task, the participants had to make saccadic eye movements to visual targets at various locations on the screen (Dell U2412M, 1,920 × 1,200 pixels, pixel pitch 0.27 mm). In each trial a central fixation dot (0.5 deg in diameter) was presented at the center of the screen for a random duration of 1,000–1,600 ms. Then the fixation spot was extinguished and the peripheral target (0.5-deg diameter) was presented at a pseudo random location for 1,000 ms. The targets were presented at five different eccentricities (3, 6, 9, 12, and 15 deg). For eccentricities up to 12 deg, targets were presented in 12 different directions (0:30:330 deg), and the targets at 15 deg eccentricity were presented in six different directions (0, 45, 135, 180, 225, and 315 deg). This resulted in a total of 54 trials at each head position. Participants were instructed to fixate the center of the targets as accurately as possible. Stimulus presentation was done with custom Matlab software using the Psychophysics Toolbox (Brainard, 1997; Pelli, 1997). This stimulus software ran on the same laptop as the stereo eyetracking program. The EyeLink toolbox for Matlab (Cornelissen, Peters, & Palmer, 2002) was used for communication with the EyeLink computer.

Offline analysis of the data was done in Matlab. The sampling rate of the cameras of the stereo eyetracker was not fixed, primarily because occasional redetecting of the eyes takes time (Fig. 2). If the eye positions are known, each camera can track stably at ~ 350 Hz. In the present experiments, the average sampling rate for a given camera was 299 ± 29 Hz (range 212–336 Hz). Because the cameras run asynchronously, the raw data of each camera were interpolated at the timestamps at which the other camera captured an image of the eyes. This resulted in a final gaze signal with an average refresh rate of 510 ± 92 Hz (range 349–660 Hz) across the different conditions.

For one participant we could not collect data for one of the nine head positions due to technical problems. In addition, for one participant we could track only one eye with the EyeLink for one of the head positions, and for another participant we could track only one eye with the stereo tracker for five head positions.

### Fixation analysis

Because at each head position different gaze angles are needed in order to fixate a target at the same screen location, we used the POG estimates for the fixation analysis. This facilitates comparison of the results at different head positions. The POG estimates in mm on the screen (Eq. 10) were converted into POG estimations in degrees. These POG estimations, in degrees, reflect the orientation of an imaginary eye placed on the *z*-axis of the stimulus coordinate system located 65 cm from the screen that is looking at the same point on the screen as the measured eye.

For each target the mean fixation location for the EyeLink and the stereo eyetracker was calculated by taking the average point of gaze during an 80-ms fixation window that started 20 ms after the end of the saccade. If a corrective saccade was made, we took an 80-ms fixation window that started 20 ms after the end of the corrective saccade. Saccades were detected on the basis of the calibrated EyeLink data with custom software. The detection of the saccade onsets and offsets was based on an eye velocity threshold criterion of 45°/s. All saccade markings were visually checked to exclude saccades in which blinks or other artifacts were present. Only trials without missing samples or artifacts during the fixation window for both eyetrackers were included in the analysis. At the central head position, an average of 8 ± 7% of the trials had to be excluded per participant due to artifacts or missing samples from either the stereo tracker and/or the EyeLink. Overall, 13 ± 18% of the trials had to be excluded for each block of 54 trials per head position.

Subsequently, for each eyetracking system and for each head position and each measured eye, the mean absolute error (MAE) between the targets and the fixation positions was calculated for the horizontal POG and the vertical POG separately to assess the accuracy of both systems. To evaluate the precision of the eyetrackers we adopted the two most commonly used measures of precision for eyetracking systems (Holmqvist et al., 2011): the standard deviation (*SD*) and the root mean squared angular displacement (RMS[s2s]) of the samples in the 80-ms fixation window.

### Saccade analysis

We analyzed the kinematics of the evoked saccades at the central head position in order to assess the dynamic properties of the two eyetracking systems. For this analysis we used the estimated gaze angles instead of the point on gaze (POG) on the screen. For the EyeLink we converted the head-referenced eye position estimates to eye rotation angles in degrees and for the stereo eyetracker we used the corrected gaze angles (Eq. 12). The data from the stereo eyetracker were resampled at the timestamps of the EyeLink samples using linear interpolation to obtain a fixed sample rate for subsequent filtering. Following the recommendations of Mack et al. (2017), we used a low-pass Butterworth filter (8th-order, 40-Hz cutoff). Zero-phase filtering was applied to avoid phase-distortion (using the function filtfilt, Matlab 2016b). Both the corrected gaze angles from the stereo tracker and the eye rotation angles from the EyeLink were passed through this filter. Subsequently, the filtered horizontal and vertical components were differentiated with respect to time by calculating the intersample difference in gaze angle (using the function gradient, Matlab 2016b) and dividing this difference by the inter-sample interval (2 ms). From this the vectorial eye velocity was computed using the Pythagorean equation.

The amplitude and the peak velocity were determined for the two systems for each first saccade in a trial. Subsequently, we determined the relation between saccade amplitude and peak velocity—that is, the main sequence (Bahill, Clark, & Stark, 1975). We fitted an exponential function (see, e.g., Goossens & Van Opstal, 1997) through the amplitude–peak velocity relation for the two systems for each participant:

13 $$ {v}_{\mathrm{peak}}={v}_0\left[1-\exp \left(-\frac{Amp}{Amp_0}\right)\right], $$

where *v*_peak_ is the peak velocity (in degrees/second), *Amp* is the saccade amplitude (in degrees), *v*_0_ is the saturation level (in degrees/second), and *Amp*_0_ is a shape parameter.

## Results

**Fig. 6:**
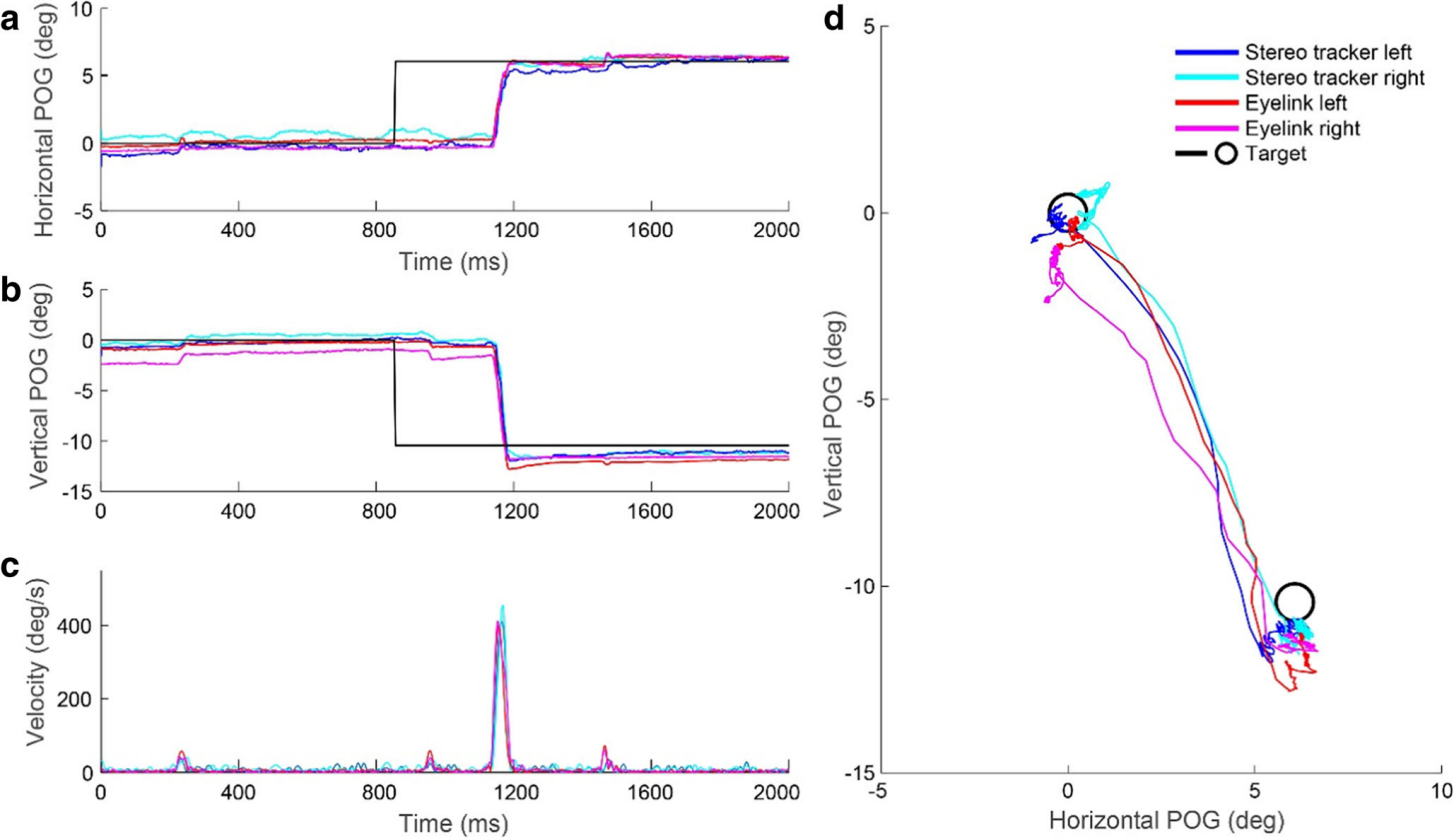
Simultaneous records of both eyetracking systems during one trial. (A) Horizontal POG estimations as a function of time. (B) Vertical POG estimations as a function of time. (C) The corresponding vectorial eye velocity traces, calculated after applying a Butterworth filter (8th-order, cutoff 40 Hz) to the position data. (D) 2-D trajectories of the POG estimations. The results for both eyes are shown. The target positions are plotted as black circles

The result of the simultaneously recorded horizontal and vertical POG estimations in degrees (see the Method section) by both eyetracking systems for one trial are presented in Figs. 6A and 6B. Figure 6C shows the corresponding 2-D trajectories of the POG estimations. In this example the accuracies of the two systems appeared to be comparable, whereas the precision of the EyeLink appeared to be slightly better. Furthermore, the vectorial eye velocity profiles, computed after applying a low-pass Butterworth filter (8th-order, 40-Hz cutoff) to the position data, were very similar for the two systems (Fig. 6C). Additional examples at a larger scale are presented in Supplement 3.

**Fig. 7:**
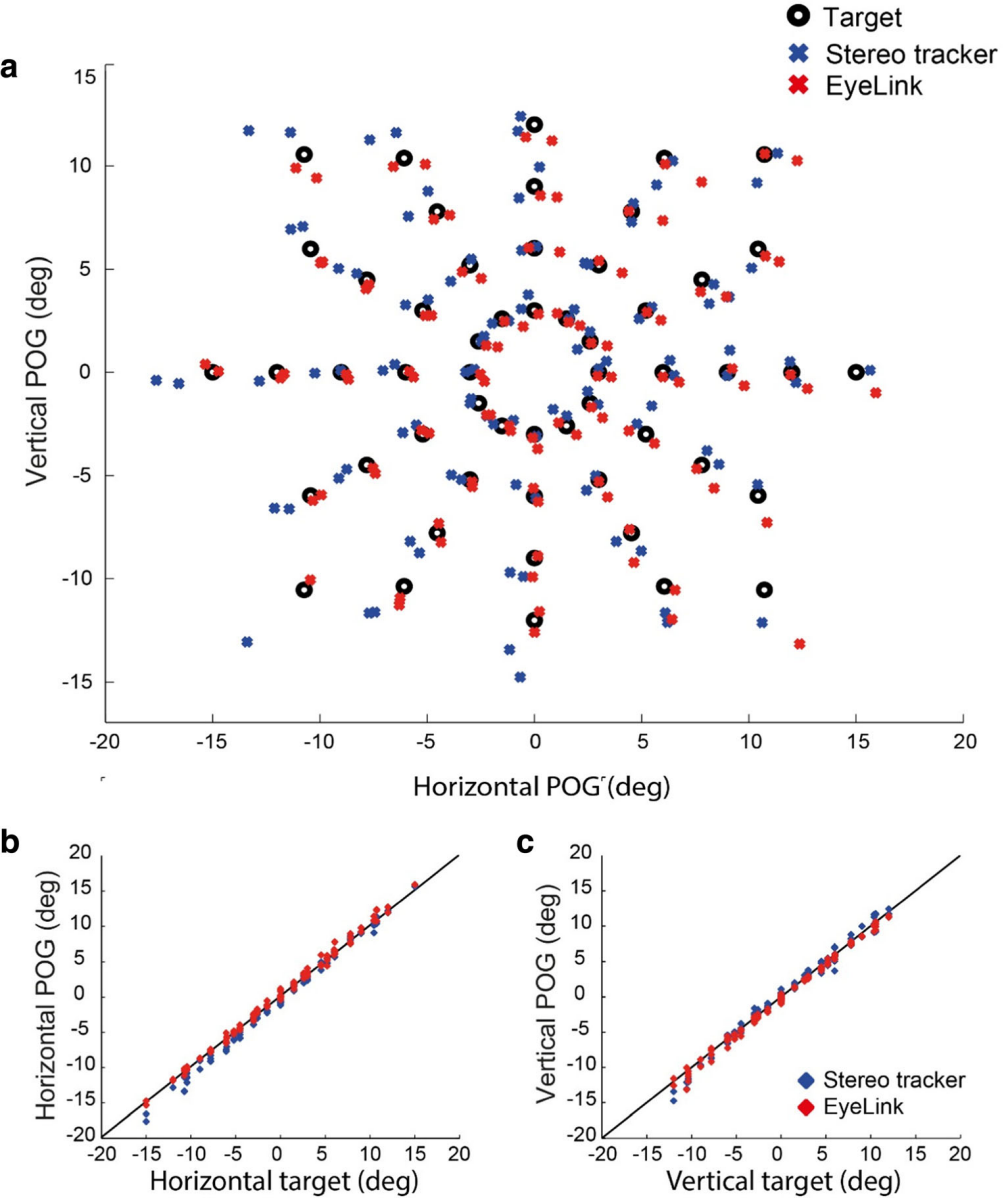
Fixation data from one participant at the central head position (both eyes). (A) 2-D target locations and the corresponding 2-D POG estimations from the EyeLink and stereo eyetracker. (B) Horizontal POG estimates plotted against the horizontal target location. (C) Vertical POG estimates plotted against the vertical target location

Figure 7 shows the results of the fixation analysis for both systems for one participant at the central head position. In general, the POG estimations obtained with the EyeLink and the stereo eyetracker were close to each target (Fig. 7A). As can be seen in Figs. 7B and 7C, throughout the measured range there was a good correspondence between the locations of the targets and the estimated POGs for both systems.

**Fig. 8:**
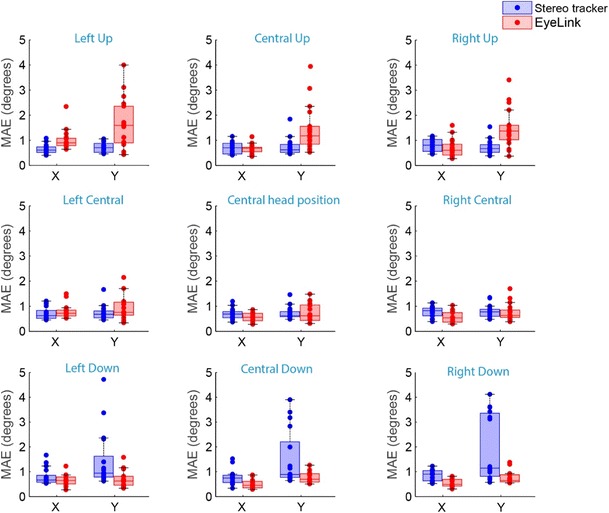
Box plots showing the accuracies of the EyeLink and the stereo eyetracker for the nine different head locations. The mean absolute errors (MAE) for all individual eyes are superimposed (dots)

The accuracies of the EyeLink and the stereo eyetracker are displayed for all participants and all head positions in Fig. 8. At the central head position the accuracy of both systems was typically better than 1 deg. The average accuracy of the stereo eyetracker at the central head position was 0.69 ± 0.21 deg horizontally and 0.73 ± 0.24 deg vertically, as compared to 0.56 ± 0.18 deg horizontally and 0.73 ± 0.37 deg vertically for the EyeLink.

When the head was translated ± 5 cm horizontally with respect to the central head positions, the accuracies of both systems remained similar to their accuracies at the central head position. The average accuracy for the three central head positions was 0.73 ± 0.22 deg horizontally and 0.75 ± 0.26 deg vertically for the stereo eyetracker, and 0.63 ± 0.25 deg horizontally and 0.80± 0.40 deg vertically for the EyeLink.

However, as can be seen in Fig. 8, vertical head translations affected the accuracies of both eyetracking systems. When the head was translated 3 cm upward from the central head position, the vertical gaze estimations of the EyeLink became less accurate. The average accuracy for the three upper head positions was 0.78 ± 0.36 deg horizontally and 1.53 ± 0.87 deg vertically for the EyeLink. For the stereo eyetracker the upward head translation did not reduce the accuracy, with an average accuracy of 0.71 ± 0.23 deg horizontally and 0.72 ± 0.27 deg vertically. The opposite effect was observed when the head was translated 3 cm downward. In that case, the accuracy of the EyeLink remained stable, with average accuracy of 0.56 ± 0.20 deg horizontally and 0.74 ± 0.28 deg vertically, while the vertical estimates of the stereo eyetracker became less accurate, with average accuracy of 1.62 ± 1.19 deg vertically and 0.80 ± 0.29 deg horizontally. Although these translations of the head resulted in average errors of > 3 deg for some participants, this did not occur for all participants. Two participants had reduced accuracies after head translations in both systems, whereas one participant had reduced accuracies for the EyeLink, and one participant had reduced accuracies for the stereo tracker.

**Fig. 9:**
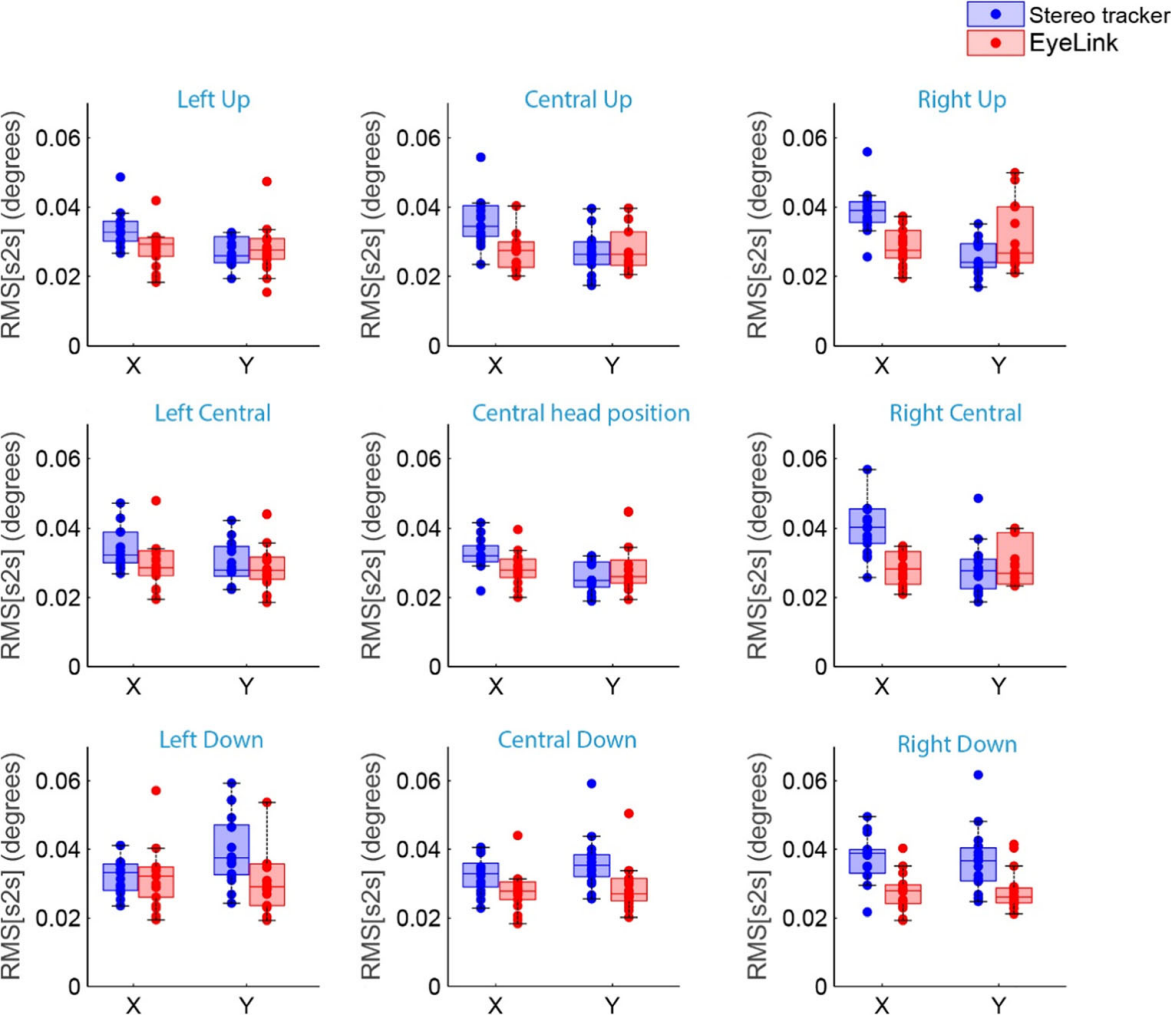
Box plots showing the intersample precision (RMS[s2s]) of the eyetrackers for the nine different head locations. The intersample precisions (RMS[s2s]) for all individual eyes are superimposed (dots)

To evaluate the precision of the eyetrackers, we determined the RMS[s2s], which is an indication of the intersample noise. The results of the RMS[s2s] analyses for the nine different head positions are presented in Fig. 9. The average RMS[s2s] across all head positions was 0.04 ± 0.007 deg horizontally and 0.03 ± 0.008 deg vertically for the stereo tracker and 0.03 ± 0.009 deg both horizontally and vertically for the EyeLink.

**Fig. 10:**
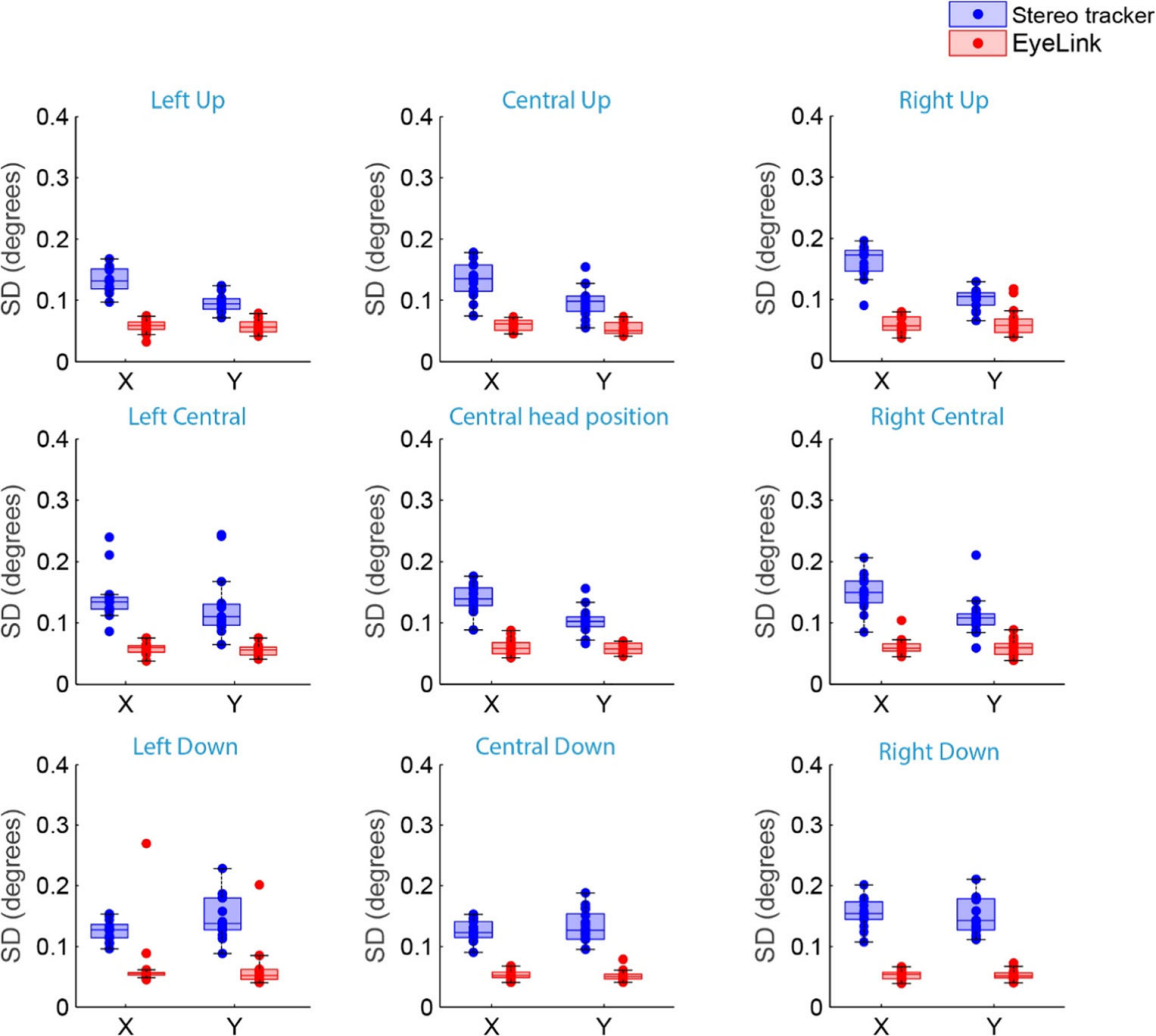
Box plots showing the standard deviations (*SD*) of the data samples during fixation, as a measure of the precision of the two eyetrackers for the nine different head locations. The *SD*s for all individual eyes are superimposed (dots)

In addition, we calculated the standard deviations (*SD*s) of the POG estimations during the 80-ms fixation window. This precision measure indicates how dispersed the samples were from the mean fixation position. The results for the *SD*s at the nine different head positions are presented in Fig. 10. The average *SD*s across all head positions were 0.14 ± 0.02 deg horizontally and 0.10 ± 0.02 deg vertically for the stereo tracker, and 0.06 ± 0.01 deg horizontally and 0.06 ± 0.01 deg vertically for the EyeLink.

**Fig. 11:**
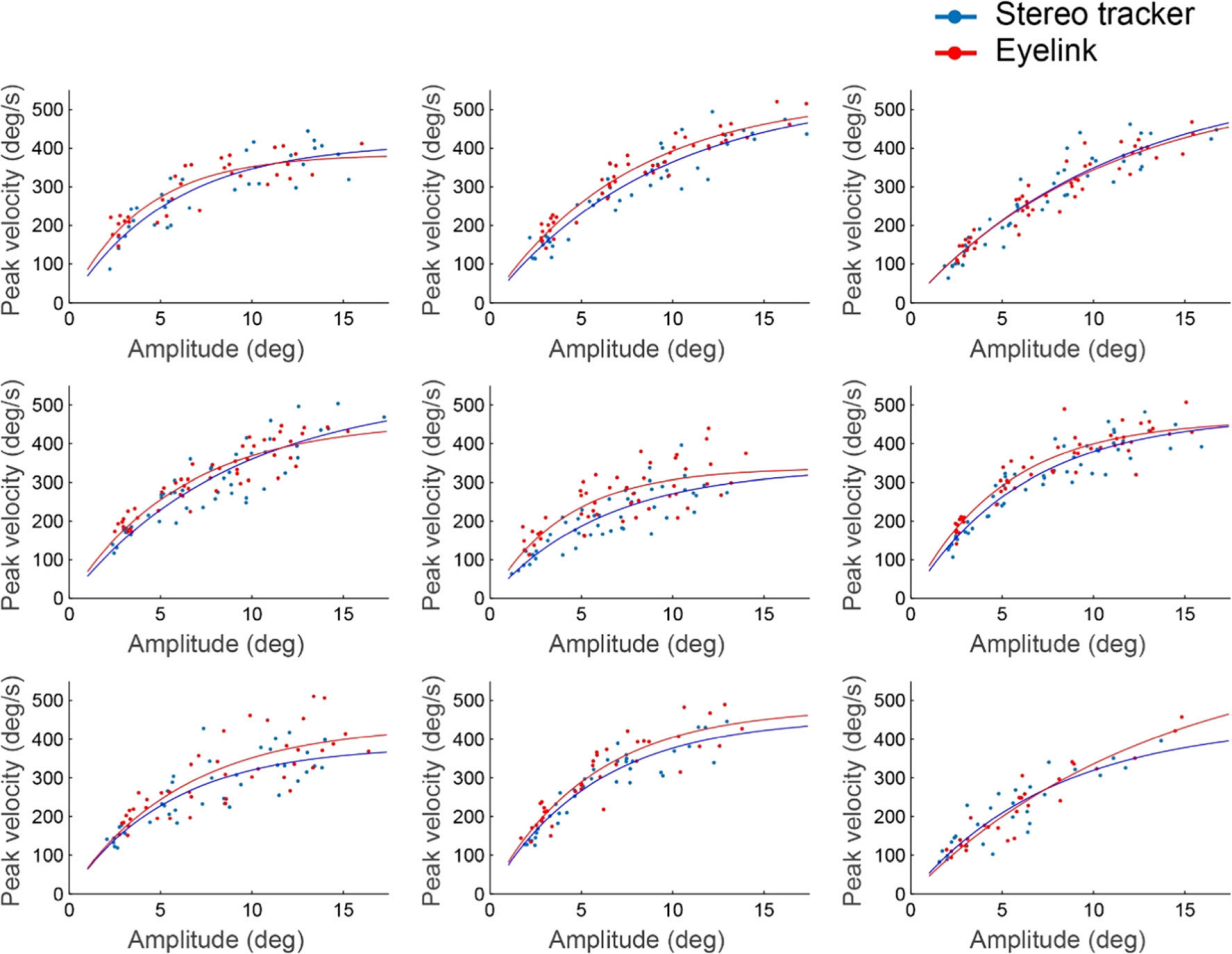
Relationship between the amplitudes of the saccades and their peak velocities as measured with the two systems. Each panel presents the results for the left eye of one participant at the central head position. Each point represents one saccade. The lines represent the fits of the main sequence (Eq. 13)

The main sequence relationship between saccade amplitude and peak velocity (Eq. 13) was determined independently for each system. The main sequence relationship for each of the nine participants at the central head positions is presented in Fig. 11. For both systems, the relationships were in line with those reported previously for visually guided saccades (Smit, Van Gisbergen, & Cools, 1987). The fits of the main sequence relationship between amplitude and peak velocity were not significantly different for the two systems.

**Fig. 12:**
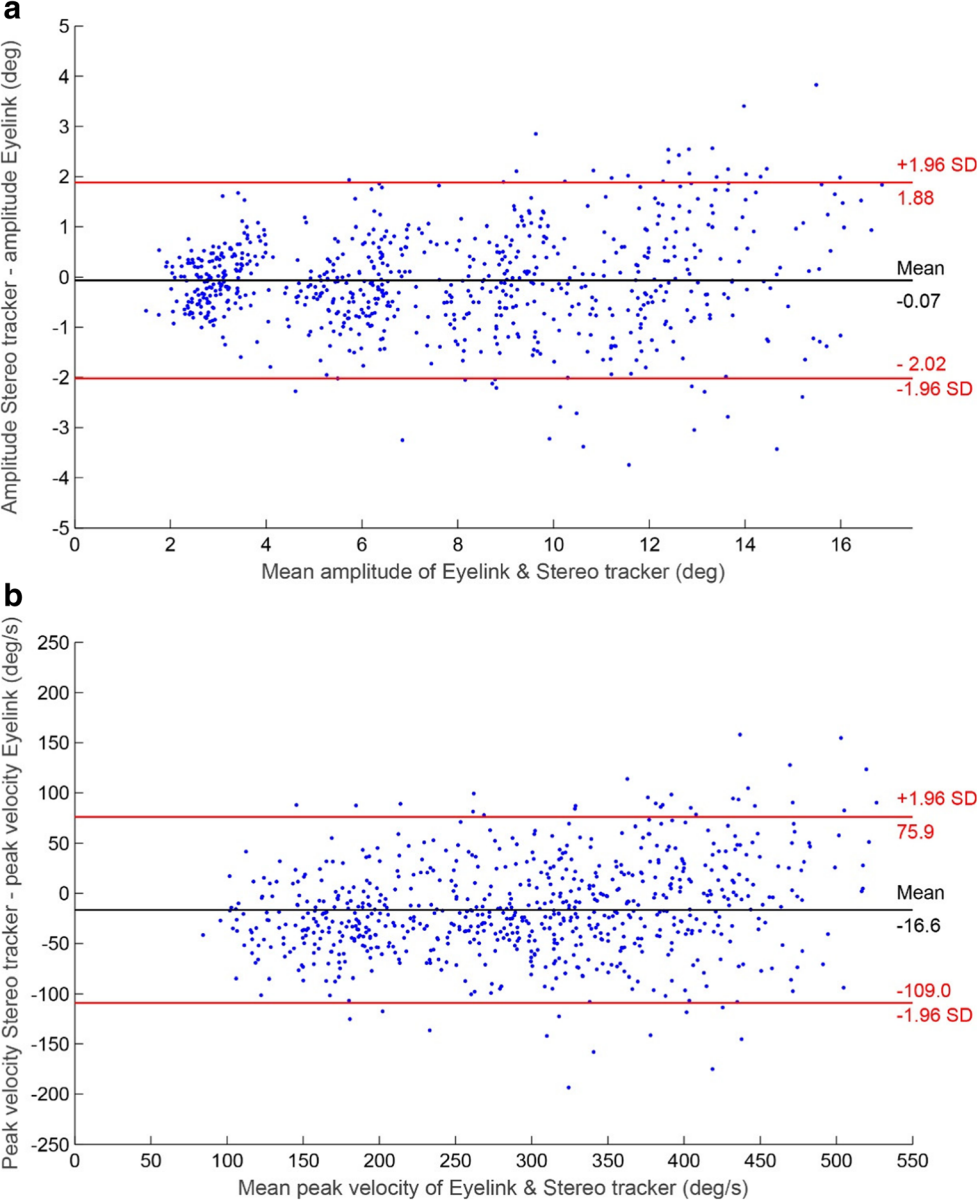
Bland–Altman plots showing the trial-to-trial differences between the stereo eyetracker and the EyeLink, with 95% confidence intervals, for (A) the saccade amplitude and (B) the peak velocity of the saccades. The data are from the central head position, pooled across eyes and participants

To analyze the correspondence between the two eyetracking systems, we constructed Bland–Altman plots for the saccade amplitude and the saccade peak velocity (Fig. 12). In these plots, the data from the central head position were pooled across eyes and participants. The average trial-to-trial differences between the two methods were small for both the amplitude (– 0.07 deg) and the peak velocity (– 16.6 deg/s), indicating that there was no significant bias in either measure. Because both eyetrackers have accuracies of ~ 0.7 deg in the horizontal and vertical directions, one may expect an *SD* of the difference in amplitude of $\sqrt{\left({0.7}^2+{0.7}^2\right)+\left({0.7}^2+{0.7}^2\right)}\approx 1.4$ deg', which predicts a 95% confidence interval (CI) of – 2.8 to 2.8 deg. However, the 95% CI of the measured difference in amplitude was smaller (– 2.02 to 1.88 deg), suggesting that the accuracies of the trackers shown in Fig. 7 are underestimated, due to inaccurate fixations from the participants.

## Discussion

We successfully developed a high-speed stereoscopic eyetracker. The average sampling rate for each camera in the present experiments was ~ 300 Hz. The interpolated gaze signal from the two cameras combined had an average refresh rate of ~ 500 Hz. The sampling rate of the two cameras is variable, because occasional redetecting of the eyes takes time. Furthermore, the two cameras track the eyes asynchronously, which causes variation in the refresh rate of the stereo tracker.

To validate the stereo eyetracker, we compared the accuracy of the point of gaze (POG) estimations obtained with the offline gaze reconstruction algorithms with the accuracy of an EyeLink 1000 Plus in remote tracking mode. The results revealed comparable accuracies (< 1 deg) at the central head positions. The EyeLink was less accurate than the specified 0.5 deg (SR Research, 2017). One of the potential causes of the reduced accuracy we encountered could be fixation disparity (disagreement between the alignment of the left and right eye). We used a monocular calibration, but measured binocularly during the experiment. Under binocular viewing conditions, the maximum amount of disparity, which still allows to fuse the input of both eyes into a single percept is about one-third of a degree (Otero-Millan, Macknik, & Martinez-Conde, 2014). We did not analyze the fixation disparity of our participants during the experiment. However, inspection of the data showed that the POG estimations of the left and the right eye were not always consistent, which indicates that fixation disparity was present. This is in line with the results from the Bland–Altman plot analysis, which suggests that part of the measured fixation errors are due to inaccurate fixations of the participants, rather than inaccuracies of the recording systems.

Vertical head displacements affected the accuracy of both eyetracking systems. The accuracy of the EyeLink was reduced for upward translations of 3 cm, whereas the accuracy of the stereo eyetracker was reduced for downward translations of 3 cm. This difference is most likely caused by the change in 3-D eye position with respect to the camera(s) and IR light source(s). Although the translations of the head resulted in average errors of > 3 deg for some participants, this did not occur for all participants. This difference in reduced accuracy of the stereo eyetracker after vertical head translations may have been caused by differences in the anatomy of the eye. Our stereo eyetracking method assumes a spherical shape of the cornea. However, in reality the cornea is slightly aspherical (Navarro, Santamaría, & Bescós, 1985). This asphericity results in biased estimates of the eye’s optical axis (Barsingerhorn et al., 2017). Due to the head translations, the asymmetry of the glints’ locations around the optic axis increased, causing a stronger effect of the asphericity. The corrections proposed in Eqs. 8–12 were quite successful in compensating for these effects, but we are the first to acknowledge that it is not immediately obvious how this is accomplished. To our knowledge, no stereo gaze reconstruction methods are available at present that actually model the asphericity of the cornea. How commercially available eyetracking systems, such as the EyeLink or the Tobii spectrum, may or may not compensate for head translations in remote tracking mode is currently undisclosed. Implementing an aspherical eye model in the stereo gaze reconstruction could, in principle, result in more accurate gaze estimations, reduced noise levels, and higher robustness against head movements. This would provide additional benefits in testing clinical populations or (young) children. In line with Guestrin and Eizenman (2010), we found that the accuracy of the POG estimates did not improve significantly if Listing’s law was included in the POG estimation (not shown). This could be due to the simplifying assumption that Listing’s plane is parallel to the vertical XY-plane, but note that for the range of eye movements that we studied (eccentricities up to 15 deg) relatively small changes in eye torsion are expected in the first place. For a larger range of eye movements, independent measurement of eye torsion (e.g., by tracking the iris pattern) would be required to better account for the effects of eye torsion.

Previous studies on stereo eyetracking only described the accuracy of the prototypes, but did not report the precision of the systems. We used two precision measures as an indication of the noise level in the gaze estimations of both systems. The intersample noise of the two systems, as indicated by the RMS[s2s], was only 0.03 deg. For the EyeLink this is in line with the technical specifications provided by the manufacturer (< 0.05 deg). As compared to other available eyetrackers the RMS[s2s] level of our stereo eyetracker is comparable, or even lower (for overviews of the RMS[s2s] of different eyetrackers, see Niehorster et al., 2017; Wang, Mulvey, Pelz, & Holmqvist, 2017). The second precision measure, the standard deviation of the samples, is an indication of the dispersion of the gaze estimates. The average *SD* of the stereo tracker was approximately twice the *SD* of the EyeLink (0.12 vs. 0.06 deg). Thus, the gaze estimates of the stereo tracker were more dispersed around the mean fixation position. This could be caused by an increased number of sources of noise due to segmentation errors and pixel noise. The EyeLink uses only the center of the pupil and the center of one glint to estimate the gaze, thus there is noise in four degrees of freedom. The stereo tracker requires two pupil centers and four glint centers to estimate the gaze, and therefore has noise in 12 degrees of freedom. Another potential source of the increased noise level in the stereo eyetracking signals is the asynchronous sampling. If one of the cameras captured the eyes at a specific timestamp, we interpolated the raw data for the other camera. If those interpolated pixel coordinates did not provide an accurate representation of the actual 3-D position and orientation of the eye, the resulting gaze estimation could deviate from the actual gaze position. This could have added variable errors to the gaze estimation of the stereo eyetracker. The results suggest that the impact of potential noise sources on the precision of the stereo eyetracker was higher for *SD* than for RMS[s2s]. Apparently, the noise reduction in Eq. 4 and the application of the median filter on the 3-D position data were more successful in removing sample-to-sample noise than in removing low-frequent variability. The exact source of this low-frequent variability is unclear. A potential source for low-frequent noise or drift in video-based eyetrackers is pupil size variations. However, the small wobbles seen in the eye position signals from the stereo tracker during fixation epochs did not correlate with the variations in pupil size (see Supplement 3).

It may be possible to optimize the filtering of the stereo eyetracking data to increase its precision. However, it is important to carefully select the filters, as they can introduce artifacts and can impact the peak velocity of saccades (Juhola, 1986; Mack et al., 2017). Another solution to decrease the level of noise could rely on an indirect mapping approach. Because the average accuracy of the stereo tracker is good, the average gaze data from an arbitrary set of fixations throughout the field of interest can in principle be used as if they were fixations at known target locations to create a polynomial mapping function between the pupil–glint vectors and gaze for each camera, or one could train a neural network to convert the raw pupil and glint coordinates from each camera into gaze estimates (see, e.g., Bremen, Van der Willigen, & Van Opstal, 2007; Goossens & Van Opstal, 2000). Such an indirect mapping approach would reduce the number of noise sources in the reconstructions, and independent gaze estimates from the two cameras could be averaged while still avoiding an elaborate calibration procedure. Furthermore, the noise level could decrease by increasing the spatial resolution of the eye images. This can be achieved by moving the cameras closer to the eyes, by using different lenses, or choosing a higher resolution setting on the camera. In the latter case, however, the temporal resolution of the eyetracker will decrease, but the other two options will restrict head motion.

Finally, analysis of the saccade kinematics revealed that the relationships between amplitude and peak velocity of the saccades—that is, the main sequence (Bahill et al., 1975)—were similar for the two systems. Moreover, a direct trial-by-by trial comparison of the amplitude and peak velocity measurements obtained from the two systems showed good agreement (Fig. 12). Therefore, the stereo eyetracker might be used to determine overall differences in the main sequence between different conditions and/or different participant groups.

Our participants did not wear glasses or contact lenses. We recommend further assessing the accuracy and precision of the stereo tracker for participants with glasses or contact lenses. The gaze reconstruction of the stereo eyetracker is based on an eye model. Therefore, the optics of glasses, especially in the case of astigmatism, could significantly impact the gaze reconstructions. Furthermore, detection of the eyes could become problematic in the case of glasses. The software uses a simple classifier to detect the eyes, the same classifier that was implemented in the ITU gaze tracker (San Agustin et al., 2010). This classifier might not be the optimal solution for eye detection: It takes relatively long to detect the eyes, and it occasionally detects the nose instead of an eye. The eyetracking software could be adapted to select the location of the eye manually if a bite-board or chin-rest can be used to stabilize the head. This would result in higher and more constant sampling rates. Moreover, other options to detect the eyes exist (for an overview, see Al-Rahayfeh & Faezipour, 2013). However, it is likely that these other methods would still be relatively slow. The easiest option would be to use a marker placed either under or above the eyes—for example, a calibrated black square on a white sticker. Not only would such a marker be easy to detect, it would also provide additional information about the 3-D position and orientation (i.e., yaw, roll, and pitch) of the head.

In conclusion, we successfully developed a high-speed (> 350Hz) stereoscopic eyetracker. The validation study showed comparable accuracies (< 1 deg) for the EyeLink 1000 Plus and our stereo system. The noise level of the stereo tracker is slightly higher. Application of the stereo eyetracker could be particularly helpful when calibration is not possible or when the experimental time is limited. In addition, it could facilitate the testing of children and clinical populations (see Supplement 4 for a proof of principle). Finally, it could be beneficial to use the stereo eyetracker in the training of naïve experimental animals, such as macaque monkeys, or in test situations in which relative gaze angles provide sufficient information (e.g., to quantify the amplitude and frequency of nystagmus).

### Author note

This research was supported by the RadboudUMC (grants to A.D.B., J.G.), Stichting Bartiméus (grant to F.N.B.), the European Union Program FP7-PEOPLE-2013-ITN ‘HealthPAC’, grant 604063 - IDP (JG), and the ODAS Foundation (grant to A.D.B., and grant 2012-35 awarded to F.N.B. and J.G.).

## Electronic supplementary material

ESM 1 (MP4 67.3 mb)

ESM 2 (MP4 145 mb)

ESM 3 (PDF 1.92 mb)

ESM 4 (PDF 988 kb)
