# Supplementary material for: Development and validation of a high-speed stereoscopic eyetracker
Source: Behav Res Methods. 2018 Mar 5;50(6):2480–97. doi: 10.3758/s13428-018-1026-7 (PMC6267515; doi:10.3758/s13428-018-1026-7)
Supplement: Supplementary file 3 — (PDF 1.92 mb) [file 13428_2018_1026_MOESM3_ESM.pdf]

Supplement 3.

Four examples of simultaneous records of both eye tracking systems during one trial for three different participants (PP 4, 8 and 6).

A. The horizontal point of gaze (POG) estimations as a function of time. POG data are expressed in degrees (See Methods). B. The vertical POG estimations as a function of time. C. The corresponding vectorial eye velocity traces (in deg/s), calculated after applying a Butterworth filter (order 8, cut-off 40 Hz) to the position data. D. Pupil size as a function of time. The pupil size data from the Eyelink are in arbitrary units, the pupil size data of the stereo tracker are in pixels. A median filter with a width of 20 samples (using the function medfilt1, Matlab 2016b) was used to filter the pupil data of the stereo tracker. Black traces indicate the location of the visual target.

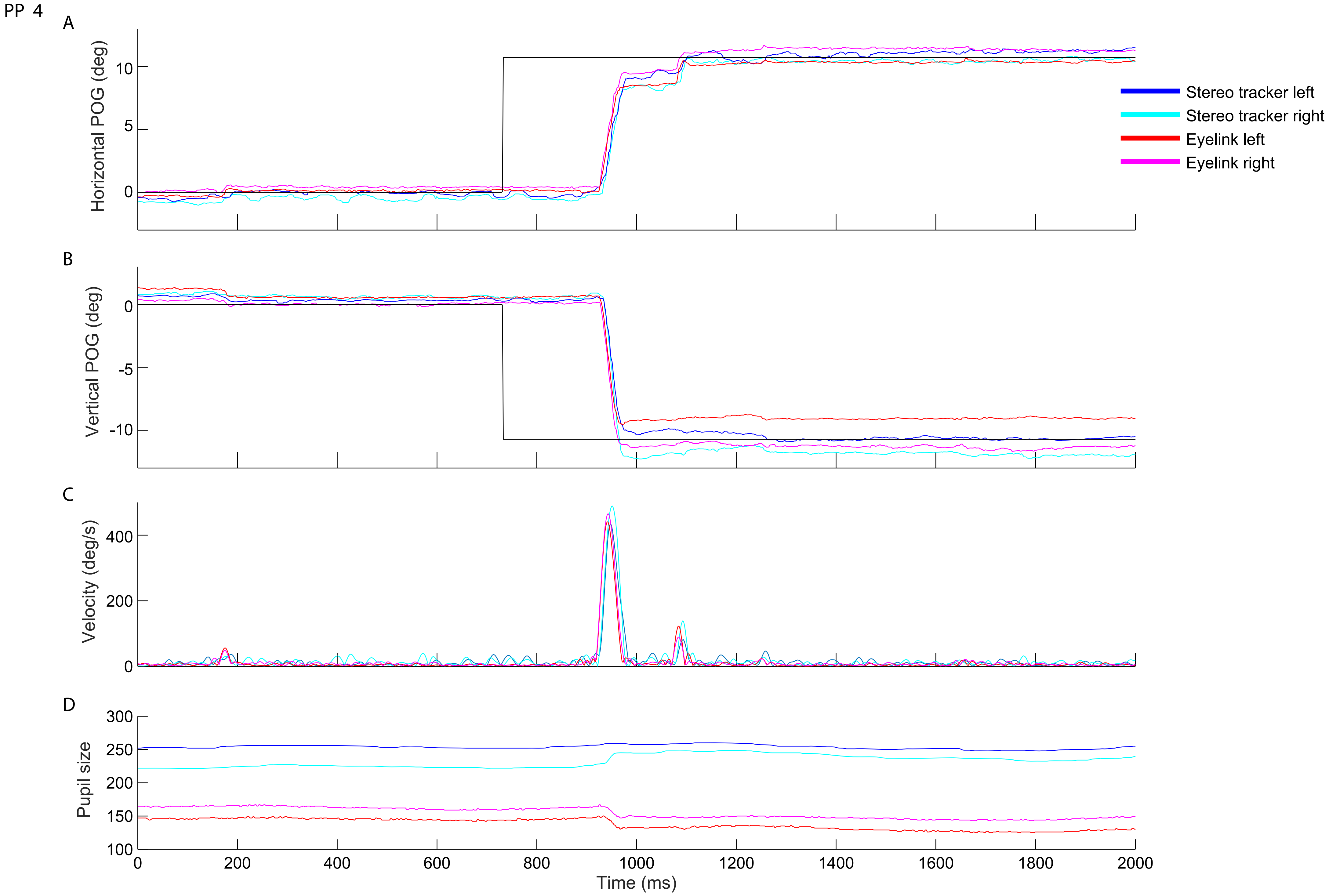

PP 4
A
Horizontal POG (deg)
10
5
0
Stereo tracker left
Stereo tracker right
Eyelink left
Eyelink right
B
Vertical POG (deg)
0
-5
-10
C
Velocity (deg/s)
400
200
0
D
Pupil size
300
250
200
150
100
0
200
400
600
800
1000
1200
1400
1600
1800
2000
Time (ms)

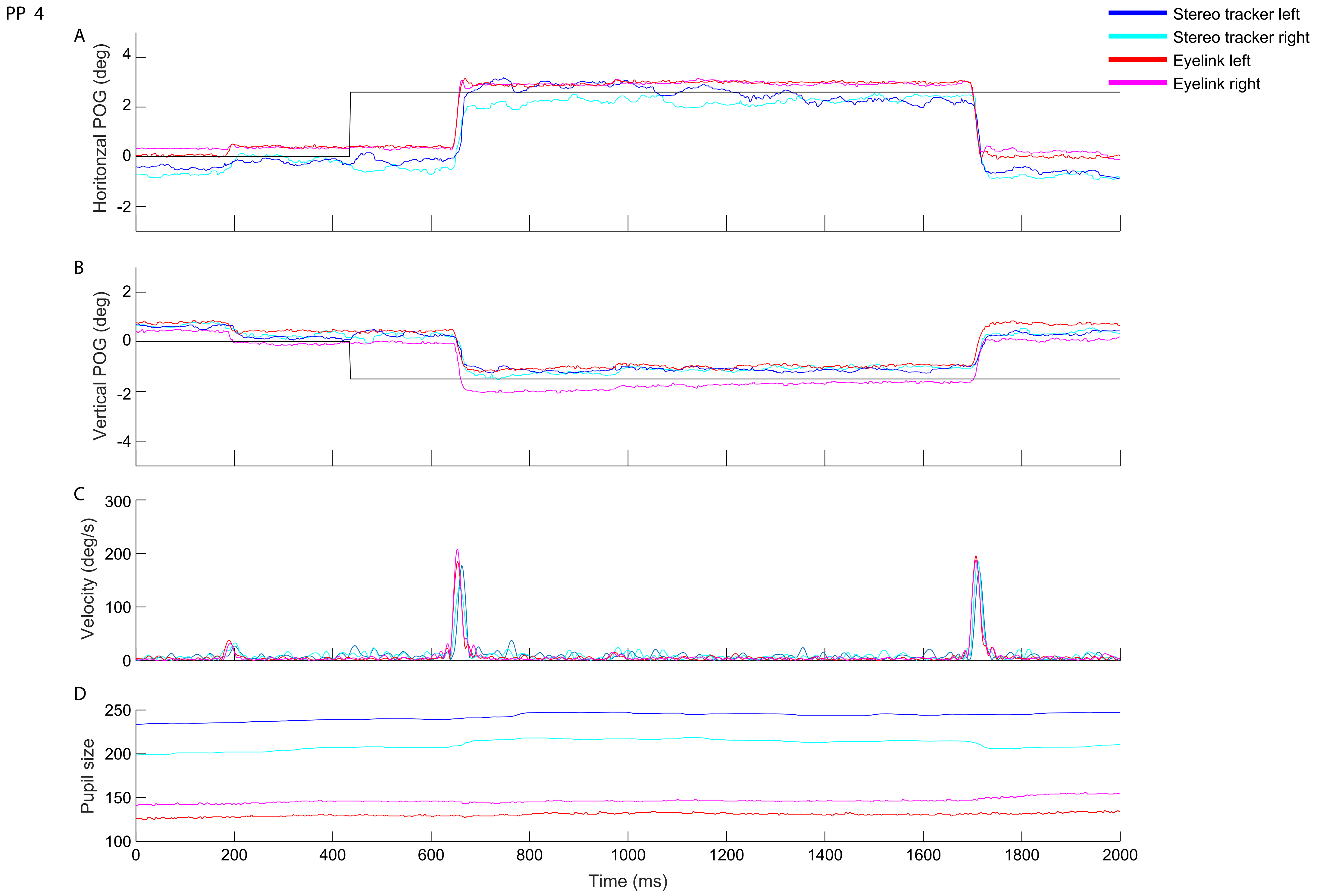

PP 4
Stereo tracker left
Stereo tracker right
Eyelink left
Eyelink right
A
Horitonzal POG (deg)
4
2
0
-2
B
Vertical POG (deg)
2
0
-2
-4
C
Velocity (deg/s)
300
200
100
0
D
Pupil size
250
200
150
100
0
200
400
600
800
1000
1200
1400
1600
1800
2000
Time (ms)

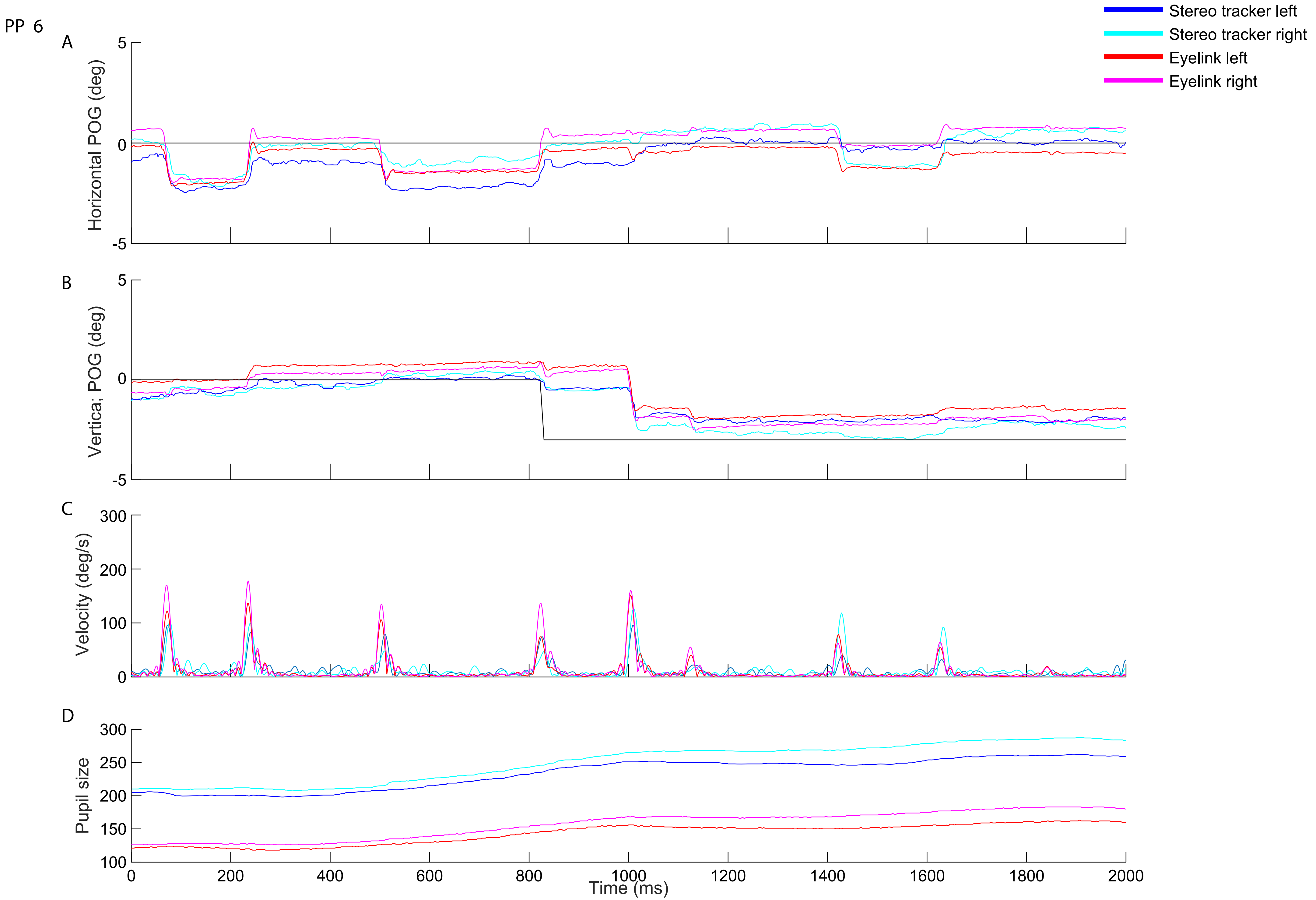
PP 6
Stereo tracker left
Stereo tracker right
Eyelink left
Eyelink right
A
Horizontal POG (deg)
5
0
-5
B
Vertica; POG (deg)
5
0
-5
C
Velocity (deg/s)
300
200
100
0
D
Pupil size
300
250
200
150
100
0
200
400
600
800
1000
1200
1400
1600
1800
2000
Time (ms)

PP 8

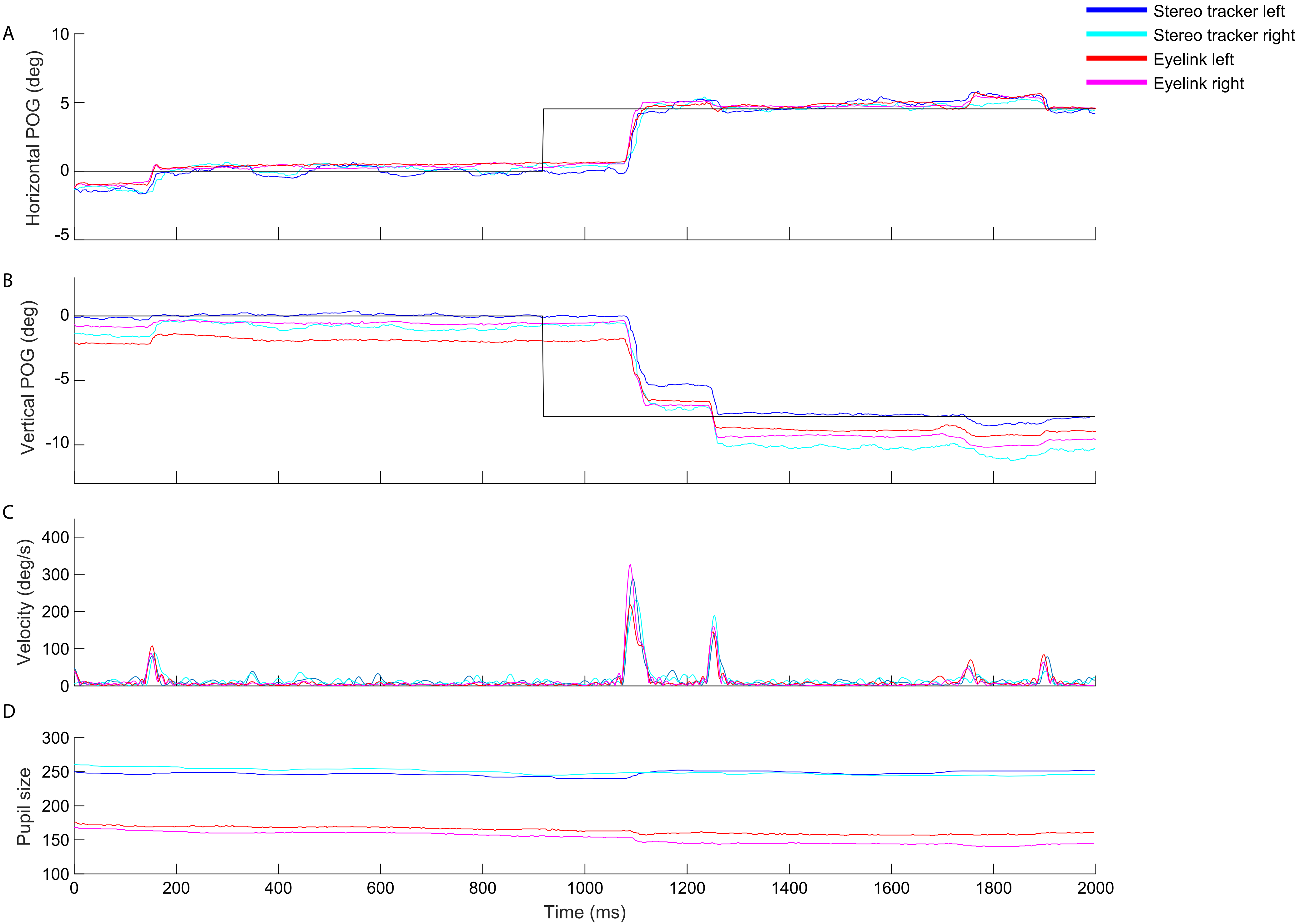

Stereo tracker left
Stereo tracker right
Eyelink left
Eyelink right
A
Horizontal POG (deg)
10
5
0
-5
B
Vertical POG (deg)
0
-5
-10
C
Velocity (deg/s)
400
300
200
100
0
D
Pupil size
300
250
200
150
100
0
200
400
600
800
1000
1200
1400
1600
1800
2000
Time (ms)
