# Supplementary material for: Development and validation of a high-speed stereoscopic eyetracker
Source: Behav Res Methods. 2018 Mar 5;50(6):2480–97. doi: 10.3758/s13428-018-1026-7 (PMC6267515; doi:10.3758/s13428-018-1026-7)
Supplement: Supplementary file 4 — (PDF 988 kb) [file 13428_2018_1026_MOESM4_ESM.pdf]

Supplement 4.

To illustrate the feasibility of applying stereo eye tracking together with a one-point calibration procedure in the target groups, we present data from two children in a head-free condition, an 11 year old with normal vision and a 10 year old with glasses and nystagmus.

A. The horizontal point of gaze (POG) estimations as a function of time. POG data are expressed in degrees (See Methods) B. The vertical POG estimations as a function of time. C. The corresponding vectorial eye velocity traces (in deg/s), calculated after applying a Butterworth filter (order 8, cut-off 40 Hz) to the position data. D. Pupil size as a function of time. The pupil size data of the stereo tracker are in pixels and were filtered with a median filter with a width of 20 samples (using the function medfilt1, Matlab 2016b). Black traces indicate the location of the visual target.

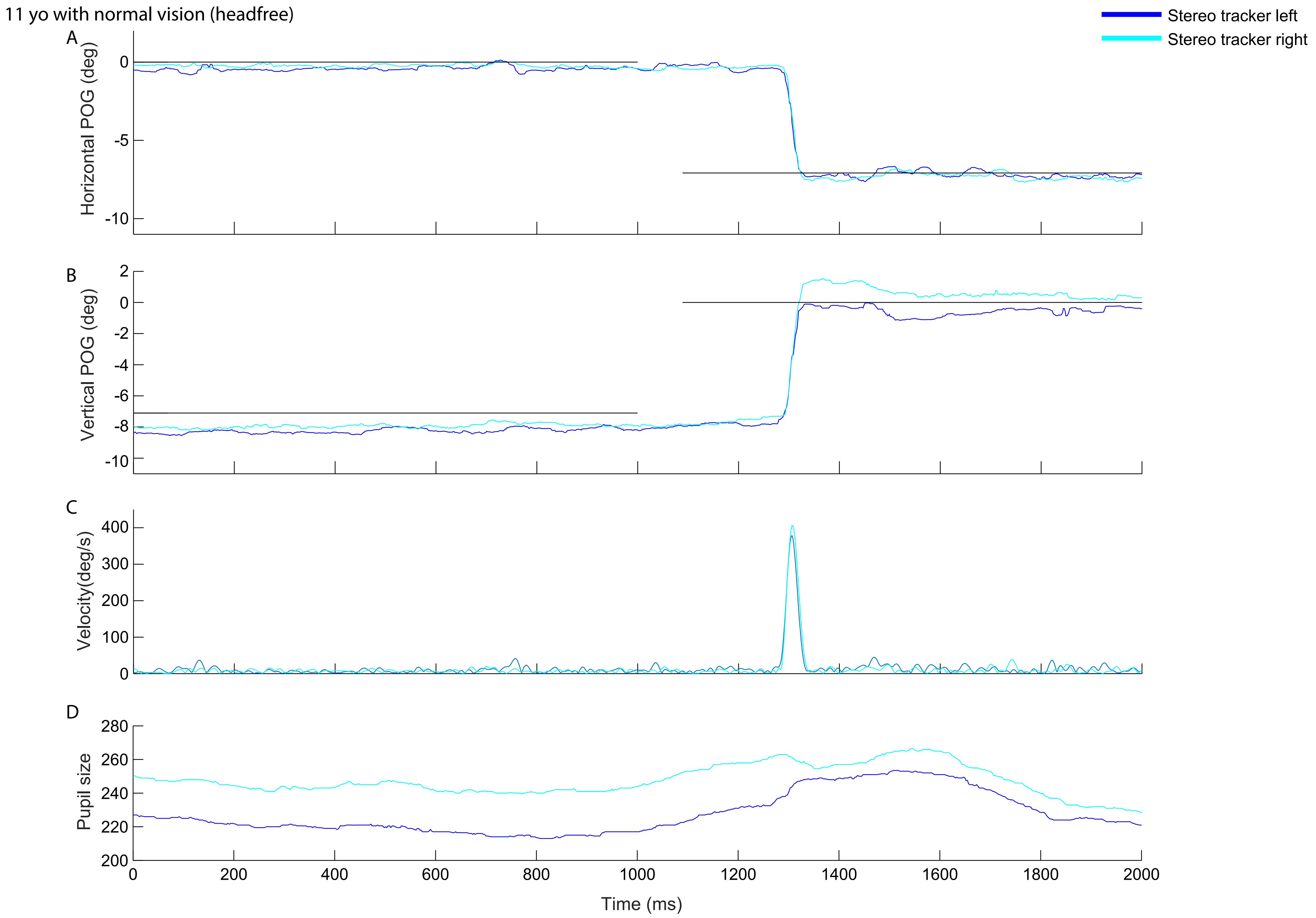
11 yo with normal vision (headfree)
Stereo tracker left
Stereo tracker right
A
Horizontal POG (deg)
0
-5
-10
B
Vertical POG (deg)
2
0
-2
-4
-6
-8
-10
C
Velocity(deg/s)
400
300
200
100
0
D
Pupil size
280
260
240
220
200
0
200
400
600
800
1000
1200
1400
1600
1800
2000
Time (ms)

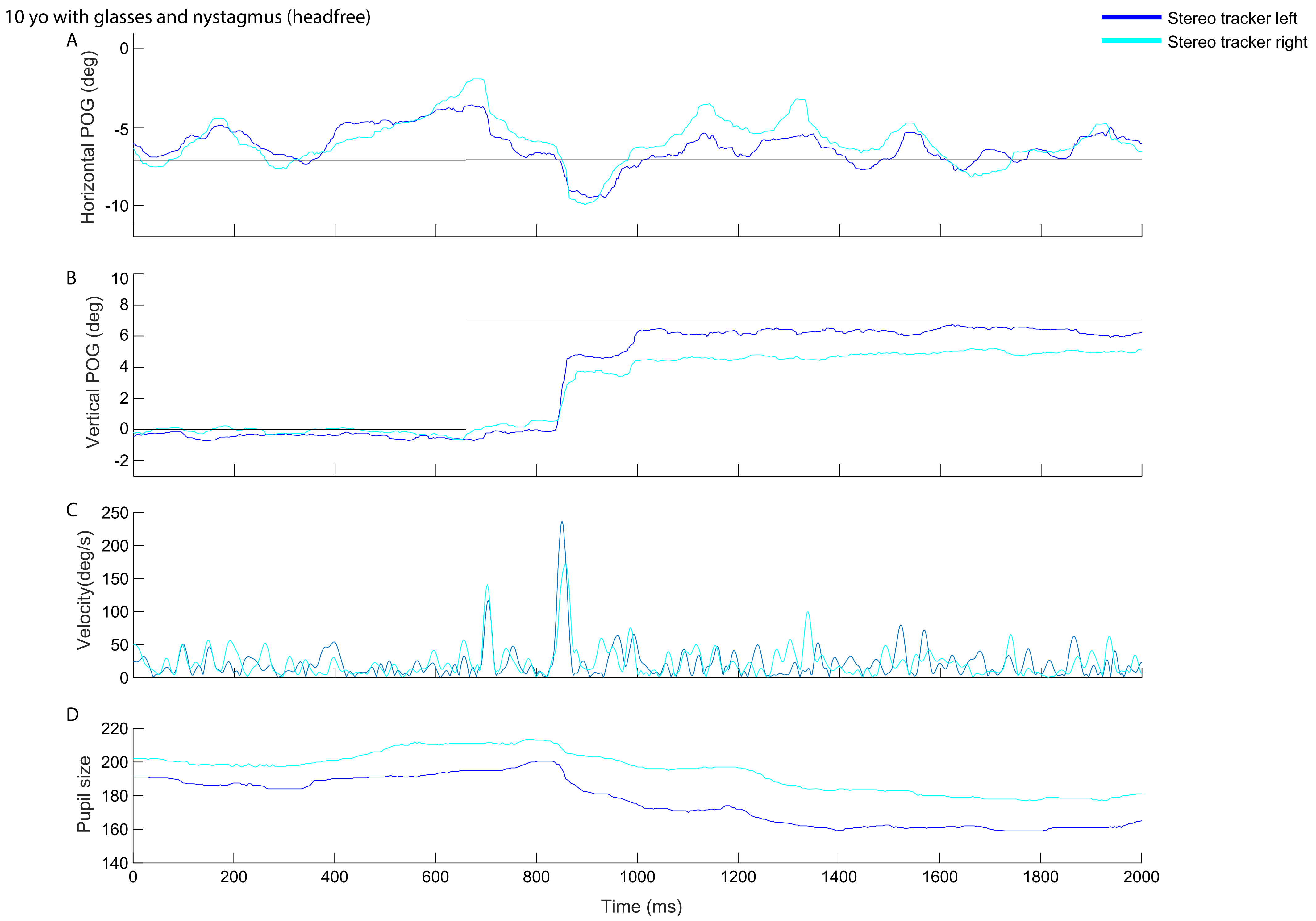
10 yo with glasses and nystagmus (headfree)
Stereo tracker left
Stereo tracker right
A
Horizontal POG (deg)
0
-5
-10
B
Vertical POG (deg)
10
8
6
4
2
0
-2
C
Velocity(deg/s)
250
200
150
100
50
0
D
Pupil size
220
200
180
160
140
0
200
400
600
800
1000
1200
1400
1600
1800
2000
Time (ms)
